# Adaptive clinical trial designs with blinded selection of binary composite endpoints and sample size reassessment

**DOI:** 10.1093/biostatistics/kxac040

**Published:** 2022-09-23

**Authors:** Marta Bofill Roig, Guadalupe Gómez Melis, Martin Posch, Franz Koenig

**Affiliations:** Section for Medical Statistics, Center for Medical Statistics, Informatics, and Intelligent Systems, Medical University of Vienna, Spitalgasse 23, 1090 Wien, Austria; Departament d’Estadística i Investigació Operativa, Universitat Politècnica de Catalunya-BarcelonaTECH, Jordi Girona 1-3, 08034 Barcelona, Spain; Section for Medical Statistics, Center for Medical Statistics, Informatics, and Intelligent Systems, Medical University of Vienna, Spitalgasse 23, 1090 Wien, Austria; Section for Medical Statistics, Center for Medical Statistics, Informatics, and Intelligent Systems, Medical University of Vienna, Spitalgasse 23, 1090 Wien, Austria

**Keywords:** Adaptive design, Clinical trial, Composite endpoint, Interim analysis, Sample size

## Abstract

For randomized clinical trials where a single, primary, binary endpoint would require unfeasibly large sample sizes, composite endpoints (CEs) are widely chosen as the primary endpoint. Despite being commonly used, CEs entail challenges in designing and interpreting results. Given that the components may be of different relevance and have different effect sizes, the choice of components must be made carefully. Especially, sample size calculations for composite binary endpoints depend not only on the anticipated effect sizes and event probabilities of the composite components but also on the correlation between them. However, information on the correlation between endpoints is usually not reported in the literature which can be an obstacle for designing future sound trials. We consider two-arm randomized controlled trials with a primary composite binary endpoint and an endpoint that consists only of the clinically more important component of the CE. We propose a trial design that allows an adaptive modification of the primary endpoint based on blinded information obtained at an interim analysis. Especially, we consider a decision rule to select between a CE and its most relevant component as primary endpoint. The decision rule chooses the endpoint with the lower estimated required sample size. Additionally, the sample size is reassessed using the estimated event probabilities and correlation, and the expected effect sizes of the composite components. We investigate the statistical power and significance level under the proposed design through simulations. We show that the adaptive design is equally or more powerful than designs without adaptive modification on the primary endpoint. Besides, the targeted power is achieved even if the correlation is misspecified at the planning stage while maintaining the type 1 error. All the computations are implemented in R and illustrated by means of a peritoneal dialysis trial.

## 1. Introduction

Composite endpoints (CEs) are frequently used in randomized controlled trials (RCTs) to provide a more comprehensive characterization of patients’ response than when using a single endpoint. For example, major adverse cardiovascular events in cardiovascular disease, where the CE includes death, stroke, myocardial infarction, or revascularization is commonly used for time-to-event endpoints (Gerstein *and others*, 2021) and binary endpoints (Cordoba *and others*, 2010). The use of CEs can also improve the power in situations where the incidence rates of the individual components are too low to achieve adequate power with feasible sample sizes and trial durations. The combination of several components into a CE provides then a solution by increasing the incidence rate of the primary endpoint. However, using CEs comes with a cost. The interpretation becomes more complex, especially when components have different effect sizes and different event probabilities. Moreover, if the treatment has only an effect in some components, the effect size of the composite will be diluted. When a CE is used as primary endpoint, regulatory agencies require to analyze in addition all components separately as secondary endpoints (FDA, 2017; EMA, 2017; Mao and Kim, 2021). In particular, it is necessary to assess the effects of the most relevant component under study. When designing a trial with a CE, sample size calculation is especially challenging since it requires the anticipation of event probabilities and effect sizes of the components of the CE as well as the correlation between them. While the marginal effect size of each component is usually known, the correlation is often not reported.

In the context of peritoneal dialysis, the binary CE major adverse peritoneal events (MAPE) has been recently proposed (Boehm *and others*, 2019). This endpoint combines three individual components: (i) peritonitis, (ii) peritoneal membrane deterioration, and (iii) technical failure; where peritonitis and peritonitis membrane deterioration endpoints are considered clinically more relevant. Given that this CE is relatively new, there is only limited data as basis for sample size calculations available. So, under which circumstances is it best to consider the CE MAPE in terms of power of the trial? Or how could we design the trial robustly to possible deviations from the anticipated correlation? In this work, we aim at addressing both questions. We propose a design in which the decision of whether it is better to consider the CE or its most relevant component as the primary endpoint is reevaluated by choosing the endpoint with the smaller required sample size. Based on this choice, the sample size is recalculated, incorporating correlation information estimated at an interim analysis if necessary. Adaptations to endpoint selection and, in particular, designs that allow adaptive modification of the primary endpoint based on interim results are discussed in the Food and Drug Administration guidance on adaptive designs (FDA, 2017, 2019). Regulatory agencies require the adaptation rule to be planned before the data become available and the use of appropriate statistical methods to ensure that the type 1 error is controlled.

In trials with multiple endpoints of interest, the testing strategy can either be based on a single endpoint (and thus consider the rest as secondary endpoints), combining all the endpoints in a CE, or considering a multiple test using all the endpoints. The choice of the primary CE based on the trial’s efficiency has been addressed by several authors. Lefkopoulou and Ryan (1993) compared the use of multiple primary endpoints to a CE by means of the Asymptotic Relative Efficiency (ARE) between the corresponding hypothesis tests. Gómez and Lagakos (2013) and Bofill Roig and Gómez Melis (2018) proposed the ARE as a method to choose between a CE or one of its components as primary endpoint for comparing the efficacy of a treatment against a control in trials with survival data and binary data, respectively. Sozu *and others* (2016) evaluated the efficiency of the trial depending on the number of endpoints considered.

Several authors have proposed different approaches to size trials with several endpoints as primary. Sozu *and others* (2010) discussed sample size formulae for multiple binary endpoints. As it is known, a major difficulty in the sample size calculation is that sometimes the required information depends on nuisance parameters or highly variable parameters. In trials with multiple endpoints, the required sample size depends on the correlation among the considered endpoints and needs to be taken into account in sample size calculations (FDA, 2017; EMA, 2017). However, the correlation between endpoints is usually unknown and often not reported in the literature which can be an obstacle for sound trial design. Several authors showed, that the correlation has a large impact on the required sample size when using multiple coprimary and composite binary endpoints (Sozu *and others*, 2010; Bofill Roig and Gómez Melis, 2019). One way to address this problem may be to consider an interim analysis to estimate unknown parameters, in particular, the correlation. Existing work in this context has mainly focused on trials with multiple endpoints. Kunz *and others* (2017) approached the sample size calculation of trials with multiple, correlated endpoints. They proposed estimators for the covariance and the correlation based on blinded data obtained at an interim analysis. Sander *and others* (2017) considered trials in which the CE and its most relevant component are two primary endpoints. They proposed an internal pilot study design where the correlation between the statistics for the CE and the most relevant component is estimated in a blinded way at an interim stage and where the sample size is then revised accordingly. Surprisingly, less attention has been given to the estimation of the correlation between the components of CEs per se and sample size reassessment in trials with primary CEs.

In this article, we propose a trial design that allows an adaptive modification of the primary endpoint based on blinded information obtained at an interim analysis and recalculates the sample size accordingly. If the primary endpoint is decided to be the CE, then the sample size reassessment incorporates the information of the estimated correlation. We focus on a two-arm RCT with a primary composite binary endpoint defined by two components, of which one is considered clinically more relevant. In Section 2, we present the problem setting and our main objectives. In Section 3, we propose the adaptive design with endpoint modification. We first introduce the decision rule used to adaptively select the primary endpoint. Then, we discuss how this decision rule is computed based on blinded data and the subsequent sample size recalculation. In Section 4, we extend the proposed design for trials with CEs of more than two components and more than two arms. In Section 5, we apply our methods to Peritoneal Dialysis trials. Furthermore, in the Supplementary material available at *Biostatistics* online, we present an R package in which the methodology has been implemented and include an additional example in the context of cardiology trials in which the R code is provided as a tutorial. We performed a blinded selection of the primary endpoint using the observed data from a conducted trial. In Section 6, we evaluate the operating characteristics of the adaptive design. We finish with a short discussion.

The R code to implement the proposed methods and reproduce the results of this article is available at https://github.com/MartaBofillRoig/eselect.

## 2. Notation, hypotheses, and trial designs

Consider an RCT designed to compare two treatment groups, a control group ($i=0$) and an intervention group ($i=1$), each composed of $n^{(i)}$ individuals, and denoting by $n=n^{(0)}+n^{(1)}$ the total sample size and by $\pi=n^{(0)}/n$ the allocation proportion to the control group. Assume two events of interest, say $\varepsilon_1$ and $\varepsilon_2$, and assume that there is one event (say $\varepsilon_1$) which is more relevant for the scientific question than the other. Let $X_{ijk}$ denote the response of the $k$th binary endpoint for the $j$th patient in the $i$th group ($i=0,1$, $j=1,...,n^{(i)}$, $k=1,2$). The response $X_{ijk}$ is $1$ if the event $\varepsilon_k$ has occurred during the follow-up and $0$ otherwise. Let $p_k^{(i)}$ represent the probability that $\varepsilon_k$ occurs for a patient belonging to the $i$th group. Let $\textrm{OR}_k = \frac{p_k^{(1)}/q_k^{(1)}}{p_k^{(0)}/q_k^{(0)}}$ denote the odds ratio for the $k$th endpoint, where $q_k^{(i)}=1-p_k^{(i)}$ ($i=0,1,k=1,2$).

Define the binary CE as the event that occurs whenever one of the endpoints $\varepsilon_1$ and $\varepsilon_2$ is observed, that is, $\varepsilon_* = \varepsilon_1 \cup \varepsilon_2$. Denote by $X_{ij*}$ the composite response defined as:

\begin{eqnarray*}
X_{ij*} &=&
\begin{cases}
1, \text{ if } X_{ij1} + X_{ij2} \geq 1 \\
0, \text{ otherwise}.
\end{cases}
\end{eqnarray*}

Let $p^{(i)}_{*}$ be the event probability of the CE, $p^{(i)}_{*}=\mathrm{P}(X_{ij*} =1)$, and ${\rm OR}_*$ be the odds ratio for the CE $\varepsilon_*$. We denote by $\hat{p}_k^{(i)}$ the estimated probability of response for the $k$th binary endpoint in group $i$, that is, $\hat{p}_k^{(i)} = \frac{1}{n^{(i)}} \sum_{j=1}^{n^{(i)}} X_{ijk} = 1 - \hat{q}_k^{(i)}$.

### 2.1. Trial design using the composite endpoint

Assume that initially the trial is planned with the CE $\varepsilon_* = \varepsilon_1 \cup \varepsilon_2$ as the primary endpoint. The hypothesis to be tested is the null hypothesis of no treatment difference in the CE $H_*: {\rm OR}_*=1$ against the alternative hypothesis of a risk reduction in the treatment group, $K_*: {\rm OR}_*<1$. We test $H_*$ using the test statistic $T_{*,n}$, given by:

(2.1) \begin{eqnarray*} \label{test:ch}
T_{*,n} &=& \frac{ \log(\widehat{{\rm OR}}_*) }{
\sqrt{ \frac{1}{n^{(0)}\hat{p}_*^{(0)} \hat{q}_*^{(0)} } + \frac{1}{n^{(1)}\hat{p}_*^{(1)} \hat{q}_*^{(1)}} }}.
\end{eqnarray*}

This statistic is asymptotically $N(0,1)$ under $H_*$ and we reject the null hypothesis if $T_{*,n}<z_\alpha$, where $z_x$ denotes the quantile of the standard normal distribution (Chow *and others*, 2017). Then the sample size needed to achieve a power of $1-\beta$ given a significance level $\alpha$ is

(2.2) \begin{eqnarray*} \label{ss_CBE}
N_*(p_*^{(0)},{\rm OR}_*) &=& \left( \frac{z_\alpha+z_\beta}{\log({\rm OR}_*)} \right)^2 \left( \frac{1}{p_*^{(0)} (1-p_*^{(0)})} + \frac{1}{\left(\frac{1-\pi}{\pi} \right) p_*^{(1)} (1-p_*^{(1)})} \right).
\end{eqnarray*}

Thus, to size a trial with a CE as primary endpoint, we need to specify the probability of an event in the CE in the control group and the odds ratio. If information on the parameters of the joint distribution of the components is available, the distribution of the CE can be derived (Bofill Roig and Gómez Melis, 2019). Specifically, the event probability of the CE in the $i$th group, $p_*^{(i)}$, is determined by the probabilities of the components, $p_1^{(i)}$ and $p_2^{(i)}$, and Pearson’s correlation coefficient between the components, $\rho$, as follows:

(2.3) \begin{eqnarray*} \label{prob_CBE}
p^{(i)}_{*} &=& 1- q_1^{(i)} q_2^{(i)} - \rho \sqrt{p_1^{(i)} p_2^{(i)} q_1^{(i)} q_2^{(i)}}
\end{eqnarray*}

The odds ratio for the CE, ${\rm OR}_*=\mathrm{OR}_*(p_1^{(0)},p_2^{(0)},{\rm OR}_1,{\rm OR}_2,\rho)$, can be expressed as function of the odds ratios ${\rm OR}_1, {\rm OR}_2$, the event probabilities in the control group, $p_1^{(0)}, p_2^{(0)}$, and the correlation $\rho$ (see the Supplementary material). Note, however, that in both cases, to compute $p^{(i)}_{*}$ (in (2.3)) and $\mathrm{OR}_*(p_1^{(0)},p_2^{(0)},{\rm OR}_1,{\rm OR}_2,\rho)$, we make the underlying assumption that the correlation between the components is the same in the treatment and control groups. Although we focus on the correlation in this work, other association measures can be used instead. In the Supplementary material, we present different association measures, such as the relative overlap and conditional probability, and establish the relationship between them and the correlation so that one can move from one to the other depending on what is easier to anticipate. More details regarding the assumption of equal correlations across arms are given in the Supplementary material.

As a consequence, the required sample size $N_*(p_*^{(0)},{\rm OR}_*)$ can be computed based on $p_*^{(0)}$, given in (2.3), and $\mathrm{OR}_*$, given in equation (1) in the Supplementary material. With a slight abuse of notation, we refer to the sample size computed by means of the components’ parameters as $N_*(p_1^{(0)},p_2^{(0)},{\rm OR}_1,{\rm OR}_2,\rho)$.

### 2.2. Trial design using the most relevant endpoint only

The null and alternative hypotheses related to the most relevant endpoint (RE) of the composite components, $\varepsilon_1$, are $H_{1}: {\rm OR}_1 = 1$ and $K_{1}: {\rm OR}_1<1$. Similar to the composite design, let $T_{1,n}$ be the statistic to test $H_1$, defined by

(2.4) \begin{eqnarray*} \label{test:eh}
T_{1,n} &=& \frac{ \log(\widehat{{\rm OR}}_1) }{
\sqrt{ \frac{1}{n^{(0)}\hat{p}_1^{(0)} \hat{q}_1^{(0)} } + \frac{1}{n^{(1)}\hat{p}_1^{(1)} \hat{q}_1^{(1)}} }}.
\end{eqnarray*}

As above, $T_{1,n}$ is asymptotically $N(0,1)$ under $H_1$, and the null hypothesis $H_{1}$ is rejected if $T_{1,n}<z_\alpha$. The sample size $N_1(p_1^{(0)}, {\rm OR}_1)$ required to achieve a power of $1-\beta$ at a one-sided significance level of $\alpha$ is given by (2.2) replacing $p_1^{(0)}$ and ${\rm OR}_1$ by $p_*^{(0)}$ and ${\rm OR}_*$, respectively.

## 3. Adaptive design with endpoint modification

### 3.1. Decision rule based on the ratio of sample sizes

We propose a trial design that allows modifying adaptively the primary endpoint based on blinded information obtained at an interim analysis or at the end of the trial. The decision rule to select the endpoint to be used as the primary endpoint chooses the endpoint with the lower estimated required sample size. Let $d(\cdot)$ denote the ratio of the required sample size for each of the designs, given by

(3.5) \begin{eqnarray*} \label{decision}
d(p_1^{(0)},p_2^{(0)},{\rm OR}_1,{\rm OR}_2,\rho) &=& \frac{N_1(p_1^{(0)},{\rm OR}_1)}{N_*(p_1^{(0)},p_2^{(0)},{\rm OR}_1,{\rm OR}_2,\rho)},
\end{eqnarray*}

where $N_1(\cdot)$ and $N_*(\cdot)$ are the sample sizes for the RE and CE introduced in Sections 2.1 and 2.2, respectively. Note that this ratio depends also on $\alpha$ and $\beta$. Now, the decision rule to select the primary endpoint is as follows: If $d(\cdot)<1$, use the most RE as the primary endpoint; if $d(\cdot)\geq 1$ the CE is chosen.

### 3.2. Estimation of the sample size ratio based on blinded data

In order to estimate the sample size ratio of the designs with the most RE and the CE, we use the blinded data obtained either at the interim analysis or the end of the trial. Specifically, we derive estimates of the event probabilities of the components in the control group and their correlation. Besides the blinded (interim) data, the estimates are based on the a priori assumptions on the effect sizes.

Suppose that the blinded analysis, using the pooled sample, is based on a sample of size $\tilde{n}$, where $\tilde{n}$ could be the total sample size initially planned ($\tilde{n}=n$) or a proportion of it used at an interim stage ($\tilde{n}=\omega \cdot n$, with $0<\omega<1$). Also, suppose that the proportion of patients assigned to the control group based on this sample is the same as the one expected at the end of the trial, that is, $\pi=n^{(0)}/n=\tilde{n}^{(0)}/\tilde{n}$, where $\tilde{n}^{(0)}$ is the sample size in the control group in the blinded data. Based on the observed responses in the pooled sample, we estimate the probabilities $p_1$, $p_2$, and $p_*$, where $p_k=\pi p_k^{(0)} + (1-\pi)p_k^{(1)}$ for $k=1,2,*$ and $\pi=n^{(0)}/n$. Assuming that the expected effects for the components (${\rm OR}_1$ and ${\rm OR}_2$) have been prespecified in advance, we obtain estimates of the probabilities of each composite component under the control group $p_1^{(0)},p_2^{(0)}$ and subsequently the estimates of the probabilities under the treatment group $p_1^{(1)},p_2^{(1)}$. Taking into account expression (2.3) and using the estimated probabilities for each composite component in each group ($\hat{p}_1^{(0)},\hat{p}_2^{(0)},\hat{p}_1^{(1)},\hat{p}_2^{(1)}$) and the estimated pooled event probability of the CE ($\hat{p}_*$), the correlation is estimated by

$$
\hat{\rho} = \frac{\hat{p}_* - \frac{\tilde{n}^{(0)}}{\tilde{n}}(1-\hat{q}_1^{(0)}\hat{q}_2^{(0)}) - \frac{\tilde{n}^{(1)}}{\tilde{n}}(1-\hat{q}_1^{(1)}\hat{q}_2^{(1)}) }{
-\frac{\tilde{n}^{(0)}}{\tilde{n}}\sqrt{\hat{p}_1^{(0)}\hat{p}_2^{(0)}\hat{q}_1^{(0)}\hat{q}_2^{(0)}}
-\frac{\tilde{n}^{(1)}}{n}\sqrt{\hat{p}_1^{(1)}\hat{p}_2^{(1)}\hat{q}_1^{(1)}\hat{q}_2^{(1)}}
},
$$

where $\hat{q}_k^{(i)}=1-\hat{p}_k^{(i)}$, and $\tilde{n}^{(i)}$ is the sample size in group $i$ in the blinded data. Based on these estimates we then compute the sample size ratio $d(\hat{p}_1^{(0)}, \hat{p}_2^{(0)},{\rm OR}_1,{\rm OR}_2,\hat{\rho})$ to select the endpoint.

**Fig. 1.**
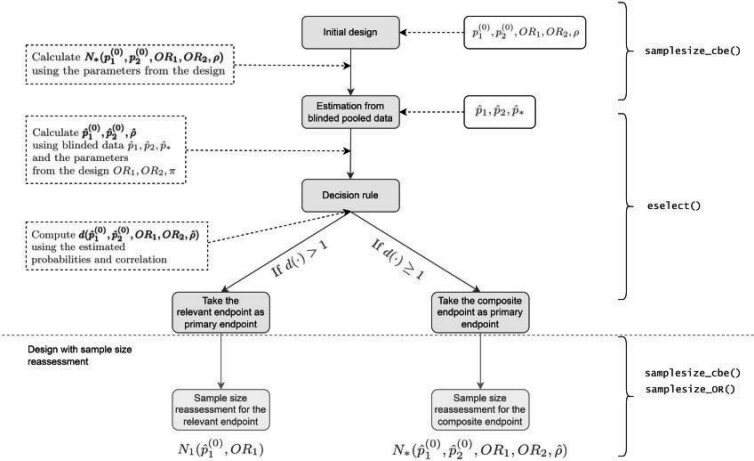
Flow diagram of the adaptive design (AD). The steps involved in adaptive design (AD). are illustrated in grey boxes. In the white boxes, there are the necessary inputs, and explanations and outputs are in dotted white boxes. The R functions to compute the corresponding steps are on the right side (see Section 5 of the Supplementary material available at *Biostatistics* online). Here $p_1^{(0)},p_2^{(0)},{\rm OR}_1,{\rm OR}_2$ denote the design parameters for the endpoints $\varepsilon_1$ and $\varepsilon_2$ and $\rho$ is the correlation between $\varepsilon_1$ and $\varepsilon_2$ used for the calculation of the initial sample size, $n$; $\hat{p}_1^{(0)},\hat{p}_2^{(0)}$ denote the estimated event probabilities in control group for $\varepsilon_1$, $\varepsilon_2$ and $\varepsilon_*=\varepsilon_1\cup\varepsilon_2$ and $\hat{p}_k$ is the estimated pooled event probability of $\varepsilon_k$ ($k=1,2,*$) based on the blinded sample $n$; $N_1$ and $N_*$ denote the sample sizes for endpoint $\varepsilon_1$ and $\varepsilon_*$ (see Sections 2.1 and 2.1), respectively; and $d(\cdot)$ is the decision function (see Section 3).

The diagram in Figure 1 exemplifies the adaptive design if initially the CE is chosen as the primary endpoint. Note that in order to calculate the initial sample size for the CE, assumptions regarding the parameters’ values determining the sample size have to be made.

### 3.3. Sample size reassessment

After the endpoint has been selected based on the estimates $\hat{p}_1^{(0)}, \hat{p}_2^{(0)},\hat{\rho}$, evaluated from the blinded data, in addition the sample size can be recalculated. When the CE is selected, the target sample size, computed from the above estimates and based on the prespecified effect sizes ${\rm OR}_1,{\rm OR}_2$, is given by $N_*(\hat{p}_1^{(0)},\hat{p}_2^{(0)},{\rm OR}_1,{\rm OR}_2,\hat{\rho})$. Because the overall sample size cannot be smaller than the number of already recruited patients, the sample size reassessment rule is given by

$$
n_a = \max\{\tilde{n}, N_*(\hat{p}_1^{(0)},\hat{p}_2^{(0)},{\rm OR}_1,{\rm OR}_2,\hat{\rho})\},
$$

where $\tilde{n}$ denotes the number of patients recruited so far.

If, in contrast, the most RE component is chosen as primary endpoint, the sample size can be reassessed to aim at a power of $1-\beta$ for this endpoint. The sample size calculation is based on the prespecified effect size ${\rm OR}_1$ and the estimated event probability $\hat{p}_1^{(0)}$. Thus, in this case the sample size reassessment rule is given by $n_a = \max\{\tilde{n}, N_1(\hat{p}_1^{(0)},{\rm OR}_1)\}$.

If the selection is made at the interim analysis, $\tilde{n}<n$ and therefore the recalculation could result in a reduction of the initially planned sample size. In contrast, if the selection is made at the planned end of the trial, $\tilde{n}=n$, the sample size can either remain unchanged or can be increased if required.

### 3.4. Considerations for choosing the timing of the interim analysis

As usual in adaptive trials, the timing of the interim analysis has to be fixed independently of the observed data and described in the trial protocol. For the proposed design, a reasonable strategy is to consider as initial sample size the minimum between the sample size for the RE and the CE assuming a correlation of $0$, that is,

$$
\tilde{n} = \min \{N_1(p_1^{(0)},{\rm OR}_1), N_*(p_1^{(0)},p_2^{(0)},{\rm OR}_1,{\rm OR}_2,\rho=0) \}.
$$

For correlation equals zero, the required sample size for the CE is the smallest (assuming that only non-negatively correlated components are possible) (Bofill Roig and Gómez Melis, 2019). Therefore, a reasonable strategy would be to fix the design as follows. First, conduct the selection of the endpoint based on blinded data after $\tilde{n}$ subjects. Then, reassess the sample size according to the rule defined in Section 3.3. If the reassessed sample size is smaller than $\tilde{n}$, stop the trial and conduct the final (unblinded) analysis of the data. Otherwise, expand the trial with further subjects as needed and conduct the final (unblinded) analysis of the selected endpoint $n_a$. The maximum sample size is bounded by the maximum sample size coming from the sample size calculation for the RE and CE assuming the largest possible correlation.

## 4. Extension to more than two components and more than two arms

In this section, we address the recursive selection of the primary endpoint for more than two components and discuss the extension to more than two arms.

### 4.1. CEs with more than two components

Consider now a trial with $K$ potential endpoints of interest. We assume that they differ in importance and can be ordered according to their importance. Let $\varepsilon_1,\ldots,\varepsilon_K$ denote the endpoints ordered by decreasing importance. Let $p_k^{(0)}$ and ${\rm OR}_k$ denote the event probabilities in the control group and the effect size for the endpoint $\varepsilon_k$ ($k=1, ...,K$). In the planning phase of the RCT, assumptions on the event probabilities, effect sizes, and correlation values are made to obtain an initial sample size estimate.

The procedure to select the primary endpoint and recalculate the sample size accordingly for $K$ components is based on the following algorithm:

**Step 1:** Compare the required sample size for the endpoint $\varepsilon_1$ and the composite of the first and second endpoints, $\varepsilon_{*,2}= \varepsilon_1 \cup \varepsilon_2$ and compute the sample size ratio based on the estimated probabilities and assumed effect sizes, $\hat{p}_1^{(0)},\hat{p}_2^{(0)},{\rm OR}_1,{\rm OR}_2$ and the estimated correlation between $\varepsilon_1$ and $\varepsilon_2$, denoted by $\hat{\rho}_{*,2}$. If $d(\hat{p}_1^{(0)},{\rm OR}_1,\hat{p}_2^{(0)},{\rm OR}_2,\hat{\rho}_{*,2}) \geq 1$, then compute the event probability and effect size of the CE, $\varepsilon_{*,2}$, denoted by $\hat{p}_{*,2}^{(0)}$ and ${\rm OR}_{*,2}$ and continue with the next step. Otherwise, select $\varepsilon_1$ and go to Step $K$.
**Steps**  $i=2,...,K-1$: Compare the efficiency of using $\varepsilon_{*,i}$ over $\varepsilon_{*,i+1}= \varepsilon_{*,i} \cup \varepsilon_{i+1}$. Compute the sample size ratio based on $\hat{p}_{*,i}^{(0)}$, ${\rm OR}_{*,i}$, computed in the previous step, and $\hat{p}_{i+1}^{(0)}$, ${\rm OR}_{i+1}$, and the estimated correlation between $\varepsilon_{*,i}$ and $\varepsilon_{i+1}$, here denoted by $\hat{\rho}_{*,i+1}$.
  If $d(\hat{p}_{*,i}^{(0)},\hat{p}_{i+1}^{(0)},{\rm OR}_{*,i},{\rm OR}_{i+1},\rho_{*,i+1})\geq1$, then compute the parameters of the CE $\varepsilon_{*,i+1}$ and go to Step $i+1$. Otherwise, select $\varepsilon_{*,i}$ and go to Step $K$.
**Step**  $K$: Reassess the sample size based on the selected endpoint.

Using this recursive method, we only need the anticipated values of event probabilities in the control and effect sizes of the components ($\varepsilon_1,\ldots,\varepsilon_K$). If the CE is selected in the step $i$, this endpoint is considered as a component for the composite considered in the next step. For this reason, the corresponding parameters are recalculated and considered as anticipated values of the components in the next iteration.

### 4.2. Trials with more than two arms

Consider a multiarmed RCT comparing the efficacy of $M$ treatments to a shared control treatment using the binary CE $\varepsilon_* = \varepsilon_1 \cup \varepsilon_2$. We test the $M$ individual null hypotheses $H_*^{(m)}: {\rm OR}_*^{(m)} =1$ against the alternative $K_*^{(m)}: {\rm OR}_*^{(m)} <1$ for each arm $m$ ($m=1, \ldots, M$), where ${\rm OR}_*^{(m)}$ denotes the odds ratio for the CE in the $m$th treatment arm.

Denoting the test statistics (2.1) to compare treatment $m$ against control by $T_{*,n}^{(m)}$, as before we have that asymptotically $T_{*,n}^{(m)}\sim N(0,1)$. We reject the null hypothesis if $T_{*,n}^{(m)}<z_{\alpha/M}$, adjusting the threshold to account for the multiplicity of treatment arms. To size the trial, suppose that the expected effect size for the components is the same in all treatment arms, that is, ${\rm OR}_k={\rm OR}_k^{(m)}$ for all $m$ ($k=1,2$). Additionally, as we did before, assume that the correlation between the components is equal across arms, $\rho=\rho^{(m)}$ for all $m$. Note that this implies that ${\rm OR}_*={\rm OR}_*^{(m)}$ for all $m$. For each individual comparison, the sample size is $N_*(p_*^{(0)},{\rm OR}_*)$ as described in Section 2, and as the trial considers a shared control the total sample size for the trial is:

$$
N_{*,M}(p_*^{(0)},{\rm OR}_*) = N_*(p_*^{(0)},{\rm OR}_*) \cdot (M- (M-1)\cdot \pi),
$$

where $\pi$ is the allocation proportion to the control group. The sample size for the multiarmed RCT can then be determined by means of the same set of parameters $(p_1^{(0)},p_2^{(0)},{\rm OR}_1,{\rm OR}_2,\rho)$.

For the most RE, the null and alternative hypotheses for treatment $m$ are $H_1^{(m)}: {\rm OR}_1^{(m)} =1$ and $K_1^{(m)}: {\rm OR}_1^{(m)} <1$. Consider the test statistics $T_{1,n}^{(m)}$ to compare treatment $m$ against control, which is asymptotically $N(0,1)$ under $H_1^{(m)}$ and reject $H_1^{(m)}$ if $T_{1,n}^{(m)}<z_{\alpha/M}$. Assuming the effect sizes to be equal across arms, ${\rm OR}_1={\rm OR}_1^{(m)}$, the total sample size for the trial would be $N_{1,M}(p_1^{(0)},{\rm OR}_1) = N_1(p_1^{(0)},{\rm OR}_1) \cdot (M- (M-1)\cdot \pi)$, where $N_1(p_1^{(0)},{\rm OR}_1)$ is the required sample size for each individual comparison.

The sample size ratio $d(\cdot)$ is then reduced to the same as used in (3.5), and the adaptive design proposed in Section 3 can then be applied analogously as for the case of a two-armed trial. Hence, if $d(\cdot)>1$, the design for testing the efficacy using the most RE(s) is chosen, otherwise the CE is, and in either case, we recalculate the sample size using the event probability and the correlation estimates. As the same effects are assumed for all arms, the same procedure can also be used to estimate the probabilities under the treatment group and the correlation. This assumption allows the estimates to be blinded and permits the selection of the primary endpoint to be the same for all arms. However, if we relax these assumptions, it could result in different selection strategies, e.g., maximizing the minimum power across all arms or partly unblinding the data (blind pooling treatment data if arms are not finishing at the same time like in multi-arm platform trials).

## 5. Motivating example in Peritoneal Dialysis Trials

Consider a trial in peritoneal dialysis with the primary endpoint MAPE, defined as the CE of peritonitis and peritoneal membrane deterioration ($\varepsilon_1$) and technical failure ($\varepsilon_2$). MAPE initially consists of three components, but we grouped peritonitis and peritoneal events together for the sake of illustration. Also, the endpoint of peritonitis and peritoneal membrane deterioration is considered as the most RE that could serve as sole primary endpoint. Table 1 summarizes the considered endpoints.

**Table 1.** Endpoints in peritoneal dialysis. Event probability and odds ratios for peritonities and peritoneal membrane deterioration and Technical failure endpoints. The event probabilities of the individual endpoints are based on results in Boehm *and others* (2019). The odds ratio for $\varepsilon_1$ and the event probability and odds ratio for the MAPE endpoint were computed assuming zero-correlations between the components of the composite endpoint

	Endpoint	Event probability	Odds ratio
Individual	Peritonities and peritoneal membrane deterioration ($\varepsilon_1$)	$0.615$	$0.52$
endpoints:	Technical failure ($\varepsilon_2$)	$0.15$	$0.66$
Composite	Major adverse peritoneal events (MAPE)	$0.703$	$0.50$
endpoint:	$\varepsilon_1 \cup \varepsilon_2$		

Boehm *and others* (2019) reported event probabilities of the individual endpoints and combinations thereof. We use these estimated event probabilities as estimates for the event probabilities in the control group at the design stage of the trial (see Table 1). We discuss the efficiency of using MAPE ($\varepsilon_*=\varepsilon_1 \cup \varepsilon_2$) over the endpoint of peritonitis and peritoneal membrane deterioration ($\varepsilon_1$) alone and illustrate the design with adaptive selection of the primary endpoint at the interim analysis and sample size reassessment.

**Fig. 2.**
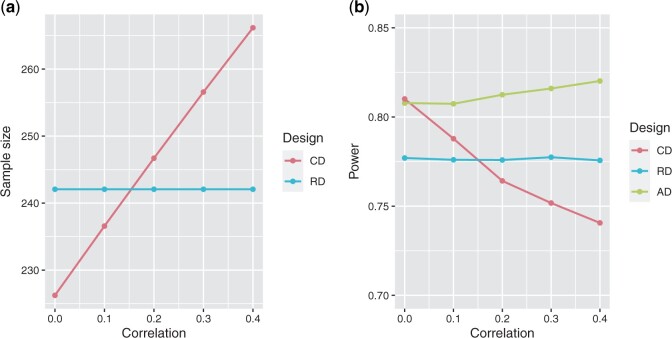
Sample size and power depending on the design and the correlation between the endpoint of peritonitis and peritoneal membrane deterioration ($\varepsilon_1$) and technical failure ($\varepsilon_2$). (a) Initial sample size when using the trial design with the most relevant endpoint of peritonitis and peritoneal membrane deterioration (RD), or with the composite endpoint Major Adverse Peritoneal Events (CD). (b) Power when using the fixed design with the most relevant endpoint of peritonitis and peritoneal membrane deterioration (RD), fixed design with the Major Adverse Peritoneal Events (CD), and the AD.

In Figure 2(a), we depict the sample size required for MAPE with respect to the correlation between $\varepsilon_1$ and $\varepsilon_2$, and the sample size if only using $\varepsilon_1$, both based on the parameters assumed at the design stage (Table 1). We can observe that the sample size increases with respect to the correlation. In Figure 2(b), we show the power of the trial when using a fixed design with the endpoint MAPE, $\varepsilon_*$, as primary endpoint, assuming that the correlation equals 0, a fixed design with the most RE $\varepsilon_1$, and when using the proposed adaptive design. We notice that the adaptive design allows to maintain the power of the trial at 0.80 and is superior to the power obtained when using the fixed design. The decision rule of the adaptive design is such that it selects the endpoint that requires the smallest estimated sample size. Furthermore, if this sample size does not result in the desired power, it is readjusted based on information from the interim analysis. So when the estimated correlation is lower than 0.2, the adaptive design typically selects the CE as primary endpoint and recomputes the sample size using the estimated correlation. When the estimated correlation is larger or equal than 0.2, then the most RE is selected and the sample size is reassessed accordingly.

## 6. Simulation study

### 6.1. Design and main assumptions

We simulate the statistical power and significance level under different scenarios and consider two-arm RCTs with two binary endpoints and parameters as given in Table 2. The correlation between the endpoints is assumed to be equal for both groups. Since the range of possible correlations depends on ($p_1^{(0)},p_2^{(0)},{\rm OR}_1,{\rm OR}_2$), scenarios in which the correlation is not within the valid range are discarded.

**Table 2.** Settings and trial designs for the simulation. Left side of the table: Parameters values used in the simulation, where ($p_1^{(0)},p_2^{(0)},{\rm OR}_1,{\rm OR}_2$) denote the parameters for the endpoints $\varepsilon_1$ and $\varepsilon_2$, $\rho$ is the correlation between the endpoints, $\omega$ is the percentage of initial sample size used for the estimation and decision rule computation, and $\alpha$ and $1-\beta$ refer to the significance level and power. Right side of the table: Trial designs considered for the simulation, including: the sample size specification for the initial calculation, whether it was based on relevant endpoint (RE) or composite endpoint (CE); and, in the case of the adaptive design, at which point in the trial the endpoint selection is made and whether sample size recalculation is considered

**Parameter settings Parameter**	**Values**	**Trial designs**		
		*All designs*	*Adaptive design*	
		**Initial sample size**	**Endpoint selection**	**Sample size reassessment**
$p_1^{(0)}$	$0.1, 0.2$	RE	At the end of the study	No
${\rm OR}_1$	$0.6, 0.8, 1$	CE	At the interim analysis	Yes
$p_2^{(0)}$	$0.1, 0.25$	RE	At the interim analysis	No
${\rm OR}_2$	$0.75, 0.8, 1$			
$\rho$	$0, 0.1, 0.2, 0.3, 0.4, 0.5, 0.6, 0.7, 0.8$			
$\omega$	$0.5$ , $1.0$			
$\alpha$	$0.05$			
$1-\beta$	$0.80$			

We compare the actual type 1 error rate and power of the proposed adaptive design with fixed designs using the RE or CE as primary endpoint. Specifically, we consider the following designs:

- Adaptive design: trial design whose primary endpoint is adaptively selected between the CE and the most RE based on blinded data.
- Composite endpoint (CE) design: trial design without adaptive modification of the primary endpoint. The primary endpoint is the CE of $\varepsilon_1$ and $\varepsilon_2$.
- Relevant endpoint (RE) design: trial design without adaptive modification of the primary endpoint. The primary endpoint is the most RE ($\varepsilon_1$).

We differentiate between two types of designs: those with selection of the components of the CE at the end of the study and those with selection at interim analysis. In the first, the selection is based on blinded data at the preplanned end of the trial, using the total sample size planned at the design stage. In the second, we select the primary endpoint based on blinded information obtained at an interim analysis after $50\%$ of the observations are available. We consider designs with and without sample size recalculation after the interim analysis.

In trials with endpoint selection at the end of the study or at interim but without recalculation of sample size, the planned sample size $n$ is calculated to have $0.80$ power to detect an effect of ${\rm OR}_1$ on the most RE at significance level $\alpha = 0.05$. We use this sample size for the three designs being compared. Therefore, the CE in this case is intended to be used only if it leads to an increase in power to the study. On the other hand, the (initial) sample size $n$ for those trials with sample size reassessment is calculated to have $0.80$ power to detect an effect of ${\rm OR}_*$ on the CE at significance level $\alpha = 0.05$, where $p_*^{(0)},{\rm OR}_*$ used for the sample size calculations are computed based on the components’ parameters ($p_1^{(0)},p_2^{(0)},{\rm OR}_1,{\rm OR}_2$) and assuming correlation equal 0. Therefore, in this case, the adaptive design serves to readjust the values anticipated in the design for the CE if the components are correlated, and to compare the efficiency of the design compared to its most relevant component, and thus to change the primary endpoint if the CE is less efficient. We summarize in Table 2 the trial designs considered for the simulation study.

For each combination ($p_1^{(0)},p_2^{(0)},{\rm OR}_1,{\rm OR}_2,\rho$), we simulated 1 00 000 trials of size $n$ according to each design (AD, CE design, and RE design). To evaluate the power, we considered the alternative hypothesis $H_1$ in which ${\rm OR}_1,{\rm OR}_2<1$ (and therefore ${\rm OR}_*<1$). We simulated based on the values assumed in the design for ${\rm OR}_1$, ${\rm OR}_2$ and the resulting ${\rm OR}_*$ computed based on the parameters ($p_1^{(0)},p_2^{(0)},{\rm OR}_1,{\rm OR}_2,\rho$). To evaluate the type 1 error rate, the same set of scenarios were considered as for the power in terms of the values used for the sample size calculation but we simulated under the global null hypothesis $H_0$ so ${\rm OR}_1={\rm OR}_2=1$ (and therefore ${\rm OR}_*=1$). The total number of scenarios is $1166$.

### 6.2. Selection at the end of the trial

As expected, for the scenarios under the alternative hypotheses the powers when using the RE design have mean $0.80$, as the sample sizes were calculated for this endpoint. The powers when using the CE design range from $0.60$ to $1.00$ with mean $0.85$. With the adaptive design, the powers take values between $0.80$ and $1.00$, and have mean $0.88$. Results are summarized in Figure 2 in the Supplementary material available at *Biostatistics* online.

**Fig. 3.**
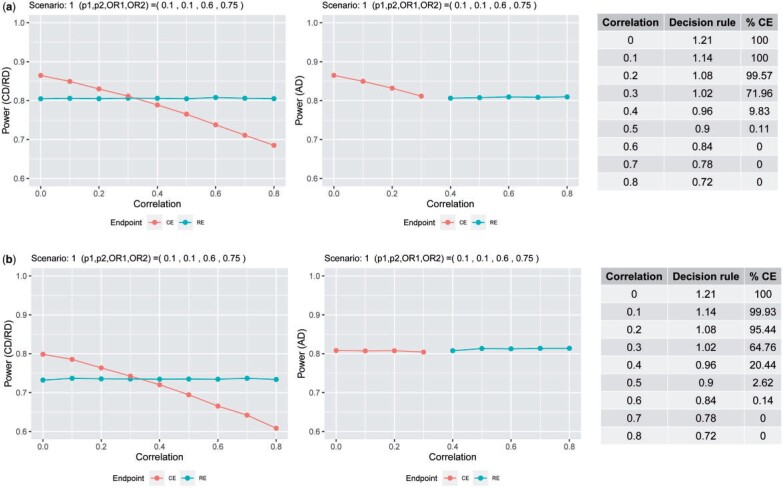
Power under composite endpoint design (CD), relevant endpoint design (RD), and adaptive design (AD) with respect to the correlation between the components. In (a), trials are initially sized to detect an effect on the RE and the ADs select the primary endpoint at the end of the trial; in (b), trials are sized to detect an effect on the CE and ADs select the primary endpoint at the end of the trial and subsequently recalculate the sample size. Tables on the right side shows the value of the decision rule computed using the parameters’ values used for the simulation and the percentage of cases in which the CE is selected as the primary endpoint. Note that for the CD and RD, the primary endpoint is the CE and RE, respectively, for the AD, the primary endpoint changes depending on the correlation.

To illustrate the properties of the adaptive design, consider a specific scenario (see Figure 3). For a given combination of ($p_1^{(0)},p_2^{(0)},{\rm OR}_1,{\rm OR}_2$), we plot the empirical power for each design (adaptive design, CE design, and RE design) for different correlations $\rho$. The colors in the power plots indicate which endpoint is optimal for the given parameters $p_1^{(0)},p_2^{(0)},{\rm OR}_1,{\rm OR}_2,\rho$. From there, we observe that when the power for the CE design is greater than $0.80$ regardless of the correlation value, the decision in the adaptive design is to use the CE. Likewise, if the CE design’s power is less than $0.80$, the RE design will be chosen. Also note that the decision rule, i.e., the ratio of sample sizes in (3.5), decreases with respect to the correlation. This is due to the sample size for CEs increasing as the components are more correlated. Indeed, for a given set of marginal parameters ($p_1^{(0)},p_2^{(0)},{\rm OR}_1,{\rm OR}_2$), the CE design is more efficient the lower the correlation. Therefore, when using the adaptive design, the decision rule chooses the CE when the estimated correlation between the components is small and chooses the most RE when the estimated power using the composite falls below $0.80$. Thus, the power of the adaptive design is always greater than $0.80$. In the Supplementary material, we plot the empirical power for each design as function of the correlation $\rho$ for all scenarios considered in the simulation. For the scenarios simulated under the global null hypothesis (i.e., ${\rm OR}_*={\rm OR}_1={\rm OR}_2=1$), all designs control the type 1 error rate at the nominal level $\alpha=0.05$.

### 6.3. Selection at the interim analysis

#### 6.3.1. With sample size reassessment

The initial sample size in these settings was computed to detect an effect on the CE, assuming uncorrelated components ($\rho=0$). For the RE design, the powers in this case range from $0.33$ to $0.85$ with mean $0.64$; and when using the CE design range from $0.60$ to $0.80$ with mean $0.72$. For the AD, in contrast, the powers have mean $0.80$ (see Figure 2 in the Supplementary material). The proposed adaptive design, therefore, ensures that the target power is achieved, either by keeping the CE as primary but correcting the correlation value assumed in the design and recalculating the sample size accordingly in the interim analysis, or by modifying the endpoint to the most RE and adjusting the corresponding sample size. To illustrate the properties of the adaptive design, we again focus on a selected scenario (see Figure 3). For the other considered cases, see the Supplementary material. We observe that when using the adaptive design, the power is always maintained at 0.80, while for the CE design it depends on the true value of the correlation and the extent to which it deviates from the correlation assumed at the design stage (which is, in our case, $\rho=0$). On the other hand, the type I error rate is as well maintained at $0.05$.

#### 6.3.2. Without sample size reassessment

When using the adaptive design with endpoint selection at an interim analysis without sample size reassessment, the observed results are slightly worse to those obtained when selecting the endpoint at the end of the study as the estimates have a higher variability. The type 1 error rate under the null scenarios investigated is again well controlled (data not shown).

### 6.4. Additional considerations

#### 6.4.1. Comparison between blinded and unblinded estimators

In this work, we proposed an adaptive modification of the primary endpoint and sample size reassessment based on parameter estimates, estimated from the blinded (interim) data. Alternatively, the event probabilities in the control group and the correlation between endpoints can be estimated using the unblinded data (but still using the a priori estimates of the effect sizes). To assess the properties of this alternative approach, we simulated adaptive trials for the above scenarios with selection at the interim analysis or at the end of the trial, and without sample size assessment. The power of the adaptive design using unblinded data is equal to or slightly higher than when using blinded data (see the Supplementary material). However, when evaluating the type 1 error, we observe that when unblinded information is used there is an inflation of type 1 error when using a conventional frequentist test as defined in Section 1. For the unblinded, the observed type 1 error rates had a maximum inflation of 0.0658 (first quartile Q1 = 0.0498, median = 0.0507, and Q3 = 0.0521), and, for the blinded, the maximum was 0.0524 (Q1 = 0.0494, median = 0.0498, and Q3 = 0.0502). The maximum type 1 error observed for the fixed designs using the CE and the RE was similar compared to the blinded case, 0.0515 and 0.0516, respectively. See Figure 3 of the Supplementary material. If the selection should be done on unblinded data in an interim analysis, more complex adaptive closed testing strategies (Bauer *and others*, 2016) have to be used and the data cannot naively be pooled over stages.

#### 6.4.2. Properties of the design if there is no treatment effect in some of the components

We additionally assessed the power of the designs in scenarios where (i) there is no effect in the most RE and (ii) there is no effect in the additional endpoint. In these settings, the adaptive design is not the most powerful design: the power of the adaptive design is between the power using only the RE and the CE designs (see the Supplementary material).

## 7. Discussion

In this article, we proposed an adaptive design that allows the modification of the primary endpoint based on blinded interim data and recalculates the sample size accordingly. The design selects either a CE or the endpoint with the most relevant component as the primary endpoint, based on the ratio of sample sizes needed in the corresponding designs to achieve a certain power. This ratio depends on the event probabilities in the control group and the effect sizes for each composite component, and the correlation between them. We presented estimators for the event probabilities and correlation based on blinded data obtained at an interim or the preplanned final analysis and proposed to use them to compute the sample size ratio. The advantage of using blinded data is that the type 1 error rate is not inflated when performing the conventional frequentist tests for the selected primary endpoint at the end of the trial. In all null scenarios investigated no substantial inflation of the type 1 error could be observed (see Figure 3 in the Supplementary material). This was expected as both the selection and sample size reassessment were based on blinded data (Posch *and others*, 2018; Kieser and Friede, 2003) and not the observed treatment effect directly. The results obtained from the proposed adaptive design are, therefore, in line with the requirements of regulatory agencies for adaptive designs with endpoint selection (FDA, 2019), since the adaptation rules for blinded endpoint selection are predefined in the design and the methods considered keep the type 1 error control.

If the selection is done at the end, we showed that the proposed design is more powerful than the fixed designs using the CE or its more relevant component as the primary endpoint in all scenarios considered in the simulation study. The simulations have shown that as long as the marginal effect sizes have been correctly specified, the power never falls below the nominal power. In addition, a reestimation of the sample size has been proposed by adjusting the sample size at the interim stage to incorporate the estimated correlation and estimated event probabilities in the control group based on the assumed effect sizes. Since the correlation between the components is rarely known and therefore not usually taken into account when sizing a trial with CEs, we want to emphasize that this sample size calculation could be useful even without adaptive modification of the primary endpoint. As in trials with CEs, the required sample size increases as the correlation increases, we proposed to start the trial assuming correlation equals zero and recalculate the sample size accordingly based on the blinded data. If sample size reassessment is not considered, then the best results are achieved when the selection of the primary endpoint is made at the end of the study due to the smaller variability of the blind estimates. However, for consistency checks and to convince external parties such as regulators, it might be reassuring to have a second independent sample, that has not been used before to determine the endpoint.

We focused on the estimation of the correlation based on blinded data but also considered estimators based on unblinded data (see the Supplementary material). We compared the operating characteristics of trial designs using blinded and unblinded correlation estimators. Power is slightly higher when using the unblinded estimator. However, it may lead to a substantial type 1 error inflation (see Section 6.4). Throughout this work, in both blinded and unblinded data cases, we assumed that correlations are equal across treatment groups. This assumption, although common, may in some cases not be satisfied. We discuss the implications of this assumption in terms of the design and interpretation, also an approach to tailor the proposed design to cases where the correlations are not equal in the Supplementary material. To allow for unequal correlations and blinded selection, one has to fix the effect size not only for the components but also for the CE. There is a trade-off by having fewer assumptions but more fixed design parameters. However, further empirical investigations are needed to evaluate how plausible it is that the equal correlation across arms assumption will not be met and the impact of different correlations on interpreting the effect of the CE.

In this article, we consider trials with large sample sizes, so derivations of sample size calculations are based on asymptotic results. In the case of trials with small sample sizes, it should be noted that smaller sizes would result in lower precision in event estimates, which could affect the variable decision and sample size recalculation. Finally, we extended the proposed design for trials with more than two groups and more than two components. Further extensions can be considered by giving greater flexibility in terms of the selection of the primary endpoint (e.g., choosing different primary endpoints according to treatment arm) and considering platform designs where the treatment arms enter and leave at different times during the trial (and therefore interim analysis also at different times). Extensions to complex designs such as those mentioned above and designs with time-to-event endpoints are open to future research.

## Supplementary Material

kxac040_Supplementary_Data

## Software

The R code to reproduce the results of this article is available at https://github.com/MartaBofillRoig/eselect.

## Supplementary material

Supplementary material is available online at http://biostatistics.oxfordjournals.org. Supplementary material includes further derivations, discussion on extensions for unequal correlations across arms, introduction of other association measures, an overview of the R package, an additional example based on a conducted cardiology trial including the R code, and other results from the simulation study.
