# Supplementary material for: Adaptive clinical trial designs with blinded selection of binary composite endpoints and sample size reassessment
Source: Biostatistics. 2022 Sep 23;25(1):237–52. doi: 10.1093/biostatistics/kxac040 (PMC10939415; doi:10.1093/biostatistics/kxac040)
Supplement: kxac040_Supplementary_Data [file kxac040_supplementary_data.zip › biosts-22064-File007.pdf]

# Supplementary material for "Adaptive clinical trial designs with blinded selection of binary composite endpoints and sample size reassessment"

Marta Bofill Roig*[1], Guadalupe Gómez Melis[2], Martin Posch[1], and Franz Koenig[1]

[1]*Section for Medical Statistics, Center for Medical Statistics, Informatics, and Intelligent Systems, Medical University of Vienna, Vienna*

[2]*Departament d'Estadística i Investigació Operativa, Universitat Politècnica de Catalunya, Barcelona, Spain*

## Contents

**1 Odds ratio for the composite endpoint** **2**

**2 Estimation of the correlation** **2**

**3 On the assumption of equal correlations across arms** **3**

3.1 Interpretation of different correlations across arms . . . 3

3.2 Adaptive design under different correlations across arms . . . 3

3.3 Tailored design for trials with different correlations across arms . . . 4

**4 Other association measures between binary endpoints** **5**

4.1 Relative overlap . . . 5

4.2 Conditional probability . . . 6

**5 `eselect` R package** **6**

**6 Example with R: Target-vessel revascularization in cardiology trials** **7**

6.1 PART A: Re-analysis of TAXUS-V with adaptive endpoint selection based on blinded data with different odd ratios for the components . . . 7

6.2 Part B: Endpoint selection assuming different effect sizes and correlations using TAXUS-V . 8

**7 Additional results simulation study** **9**

7.1 Results for designs using unblinded data . . . 11

7.2 Additional results with sample size assessment . . . 13

7.3 Additional results without sample size assessment . . . 19

*E-mail: marta.bofillroig@meduniwien.ac.at

# 1 Odds ratio for the composite endpoint

Let $X_{ij1}$ and $X_{ij2}$ denote the responses of two binary endpoints for the $j$-th patient in the $i$-th group of treatment ($i = 0, 1,\ j = 1, ..., n^{(i)}$). Denote by $X_{ij*}$ the composite response defined as

$$X_{ij*} \;=\; \begin{cases} 1, & \text{if } X_{ij1} + X_{ij2} \geq 1 \\ 0, & \text{if else } X_{ij1} + X_{ij2} = 0 \end{cases}$$

Denote by $p_1^{(i)} = \mathrm{P}(X_{ij1} = 1) = 1 - q_1^{(i)}$, $p_2^{(i)} = \mathrm{P}(X_{ij2} = 1) = 1 - q_2^{(i)}$ and $p_*^{(i)} = \mathrm{P}(X_{ij*} = 1) = 1 - q_*^{(i)}$ the probabilities of observing each endpoint in the $i$-th group. Let $\mathrm{OR}_1, \mathrm{OR}_2$ be the odds ratio for both endpoints, that is, $\mathrm{OR}_k = \frac{p_k^{(1)}/q_k^{(1)}}{p_k^{(0)}/q_k^{(0)}}$ for $k = 1, 2$. Let $\rho^{(i)}$ the correlation between $X_{ij1}$ and $X_{ij2}$ in group $i$ ($i = 0, 1$). The probability of the composite endpoint is given by

$$p_*^{(i)} \;=\; 1 - q_1^{(i)} q_2^{(i)} - \rho^{(i)} \sqrt{p_1^{(i)} p_2^{(i)} q_1^{(i)} q_2^{(i)}} \tag{1}$$

and the odds ratio for the composite endpoint, $\mathrm{OR}_*$, can be expressed in terms of the odds ratios $OR_1$ and $OR_2$, the probabilities under the control group, $p_1^{(0)}$ and $p_2^{(0)}$, and the correlation $\rho$ as follows:

$$\mathrm{OR}_* = \frac{\dfrac{\left(1+\frac{\mathrm{OR}_1 p_1^{(0)}}{1-p_1^{(0)}}\right)\left(1+\frac{\mathrm{OR}_2 p_2^{(0)}}{1-p_2^{(0)}}\right) - 1 - \rho^{(1)}\sqrt{\frac{\mathrm{OR}_1 \mathrm{OR}_2 p_1^{(0)} p_2^{(0)}}{(1-p_1^{(0)})(1-p_2^{(0)})}}}{1+\rho^{(1)}\sqrt{\frac{\mathrm{OR}_1 \mathrm{OR}_2 p_1^{(0)} p_2^{(0)}}{(1-p_1^{(0)})(1-p_2^{(0)})}}}}{\dfrac{\left(1+\frac{p_1^{(0)}}{(1-p_1^{(0)})}\right)\left(1+\frac{p_2^{(0)}}{(1-p_2^{(0)})}\right) - 1 - \rho^{(0)}\sqrt{\frac{p_1^{(0)} p_2^{(0)}}{(1-p_1^{(0)})(1-p_2^{(0)})}}}{1+\rho^{(0)}\sqrt{\frac{p_1^{(0)} p_2^{(0)}}{(1-p_1^{(0)})(1-p_2^{(0)})}}}} \tag{2}$$

# 2 Estimation of the correlation

Next, we describe two different approaches for estimating the correlation between the components of the composite endpoint ($\rho$), where the correlation is assumed to be equal in the treatment and control groups. As in the main paper, suppose we have a sample of size $\tilde{n}$, where $\tilde{n}$ could be the total sample size initially planned ($\tilde{n} = n$) or a proportion of it used at an interim stage ($\tilde{n} = p_{init} \cdot n$, with $0 < p_{init} < 1$). Also, suppose that the proportion of patients assigned to the control based on this sample is the same as the one expected at the end of the trial, that is, $\pi = n^{(0)}/n = \tilde{n}^{(0)}/\tilde{n}$ where $\tilde{n}^{(0)}$ is the sample size in the control group in the blinded data.

**Blinded approach**:

Let $p_k$ be the probability of observing the $k$-th endpoint in the pooled sample and $\hat{p}_k$ be its estimate, that is:

$$\hat{p}_k \;=\; \sum_{i=0,1} \sum_{j=1}^{\tilde{n}^{(i)}} X_{ijk}/\tilde{n} \tag{3}$$

for $k = 1, 2, *$.

Based on the observed responses in the pooled sample, we estimate the probabilities $p_1$, $p_2$, and $p_*$. Once the pooled estimates for each endpoint have been obtained, we calculate the estimated event probabilities per group. For this, we take into account that the pooled estimate over both groups is the weighted mean of the event probabilities per group, given by

$$p_k \;=\; \pi p_k^{(0)} + (1-\pi) p_k^{(1)} \tag{4}$$

and $p_k^{(1)} = (OR_k \cdot p_k^{(0)}/(1-p_k^{(0)}))/(1 + (OR_k \cdot p_k^{(0)}/(1-p_k^{(0)})))$. By using these equations, and plugging in the estimate $\hat{p}_k$ and assuming the expected effects for the endpoints 1 and 2 (say $OR_1$ and $OR_2$) pre-specified

in advance, we obtain estimates of the probabilities of each composite component under the control group, $\hat{p}_1^{(0)}, \hat{p}_2^{(0)}$, and subsequently the estimates of the probabilities under the treatment group, $\hat{p}_1^{(1)}, \hat{p}_2^{(1)}$. By this step-wise estimation procedure only blinded estimates and assumptions regarding the effect sizes are used to back calculate the event probabilities per group.

Taking into account equation (2.3) in the paper and using the estimated probabilities for each composite component in each group $(\hat{p}_1^{(0)}, \hat{p}_2^{(0)}, \hat{p}_1^{(1)}, \hat{p}_2^{(1)})$ and the estimated pooled probability of the composite endpoint $(\hat{p}_*)$, we get the following estimator of the correlation:

$$\hat{\rho} = \frac{\hat{p}_* - \frac{\tilde{n}^{(0)}}{\tilde{n}}(1 - \hat{q}_1^{(0)}\hat{q}_2^{(0)}) - \frac{\tilde{n}^{(1)}}{\tilde{n}}(1 - \hat{q}_1^{(1)}\hat{q}_2^{(1)})}{-\frac{\tilde{n}^{(0)}}{\tilde{n}}\sqrt{\hat{p}_1^{(0)}\hat{p}_2^{(0)}\hat{q}_1^{(0)}\hat{q}_2^{(0)}} - \frac{\tilde{n}^{(1)}}{\tilde{n}}\sqrt{\hat{p}_1^{(1)}\hat{p}_2^{(1)}\hat{q}_1^{(1)}\hat{q}_2^{(1)}}}$$

where $\hat{q}_k^{(i)} = 1 - \hat{p}_k^{(i)}$.

**Unblinded approach:**

Based on the observed responses in patients in the control group, we estimate the probabilities $p_1^{(0)}, p_2^{(0)}, p_*^{(0)}$. By using equation (2.3), and replacing $p_*^{(0)}, p_1^{(0)}, p_2^{(0)}$ with their estimated values, we obtain an estimate of the correlation between the Endpoints 1 and 2 in the control group, $\hat{\rho}^{(0)}$. We do the same for the treatment group, and then we obtain an estimate of the correlation in the treatment group, $\hat{\rho}^{(1)}$.

We then consider the estimator:

$$\hat{\rho} = (\hat{\rho}^{(0)} + \hat{\rho}^{(1)})/2.$$

# 3 On the assumption of equal correlations across arms

## 3.1 Interpretation of different correlations across arms

Under the null hypothesis of no treatment effect on none of the components of the composite, we assume that the distribution of the components is equal between arms. As the rationale for using composite endpoints is to evaluate if the new treatment under study reduces the probability of patients suffering from any of the events included in the composite, it is natural to assume that under the null the joint distribution between them is also the same in both arms. And hence, the assumption of equal correlations holds naturally. Under the alternative hypothesis, it could be the case that one of the components has a greater effect than the other component and that in some way this also alters the correlation between the components.

We assessed how the distribution of the composite endpoint relies on the assumption on equal correlations. For that, we looked at how the composite endpoint's distribution behaves when we fix the correlation in the control arm, $\rho^{(0)}$, and vary the correlation in the treatment arm, $\rho^{(1)}$. As the probability of the composite endpoint decreases as the correlation increases, the effect in terms of the odds ratio (in (2)) increases with respect to $\rho^{(1)}$ and hence the sample size for the composite endpoint decreases with $\rho^{(1)}$. In the scenarios assessed (data not shown), both the effect and the sample size are very sensitive to changes in correlation. Given that the objective when considering composite endpoint is to assess whether there is a reduction in patients having events considered in the composite, if the treatment effect is primarily driven by a change in the correlation between events rather than a reduction in events per se, the use of composite endpoint should be reconsidered.

## 3.2 Adaptive design under different correlations across arms

We evaluated the robustness regarding the assumption of different correlations in the proposed design with respect to the statistical power through simulations. We simulated two-arm trials as described in Section 7 of the paper. We considered two sets of parameters values for $(p_1^{(0)}, p_2^{(0)}, OR_1, OR_2)$, assumed correlation in the control arm $\rho^{(0)}$ equal to 0.2 and varied the correlation in the treatment arm $\rho^{(1)}$ between 0 and 0.5. Figure 1 shows the power of the adaptive design with respect to the correlation $\rho^{(1)}$.

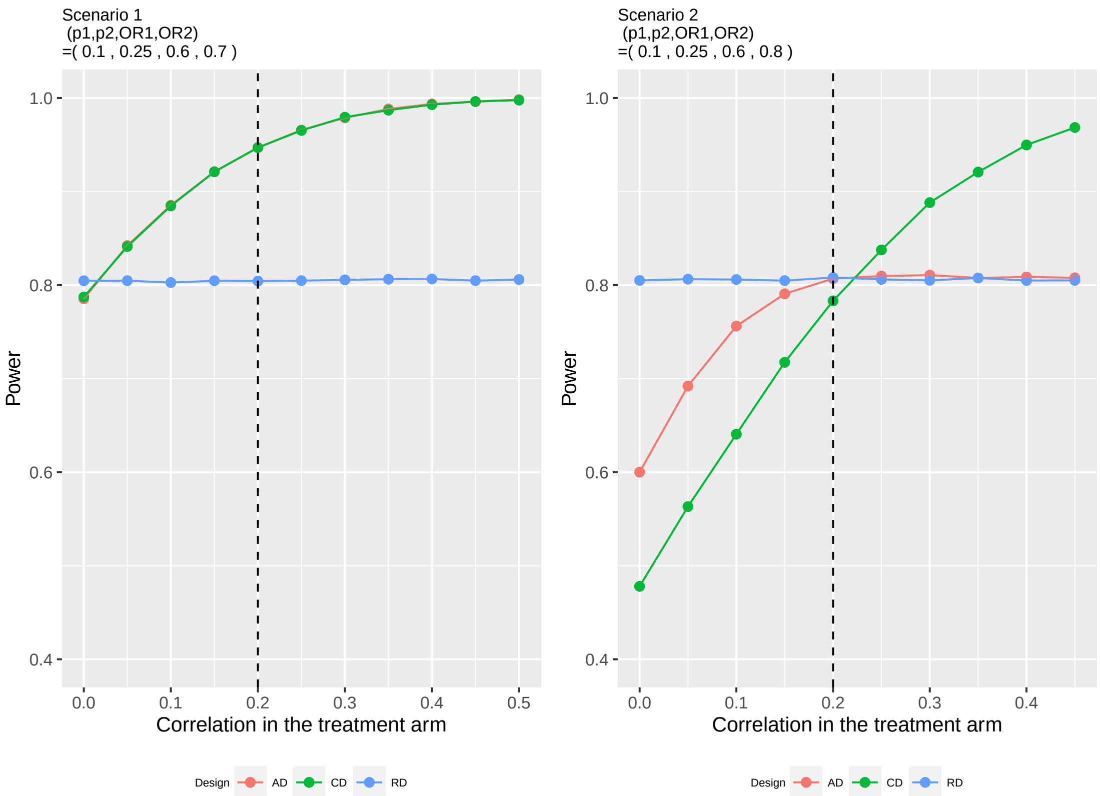


Figure 1: Power under composite design (CD), relevant design (RD) and adaptive design (AD) with respect to the correlation in the treatment arm ($\rho^{(1)}$) when the correlation in the control arm ($\rho^{(0)}$) equals 0.2. Note that red and green curves in the left panel are overlapping.

In all the cases considered, the reassessed sample size took values between the sample size computed by correctly assuming different correlations and the sample size assuming equal correlations. Hence, in terms of the decision, if for all the values of $\rho^{(1)}$ the sample size of the composite endpoint is smaller than the sample size for the relevant endpoint, the decision is not affected. As is the case in, for example, scenario 1 (left panel), where we can see that the decision is always to choose the composite endpoint and therefore both curves overlap in Figure 1. Otherwise, however, the estimated sample size in the interim stage may lead to wrong decisions and therefore the adaptive design selects an endpoint that might not lead to the most powerful trial (as is the case in scenario 2).

### 3.3 Tailored design for trials with different correlations across arms

In situations where it is anticipated that different correlations between arms may arise, one could apply an adaptive design similar to the one proposed assuming the expected effect on the composite endpoint, $OR_*$, is also anticipated in the planning stage.

The tailored adaptive procedure for different correlations is then defined by the following algorithm:

(0) Set the initial values at the design stage: $p_1^{(0)}, p_1^{(0)}, OR_1, OR_2, OR_*, \rho^{(0)}$

(1) Blinded estimation of the event probabilities and correlations: Based on the responses in the pooled sample, we estimate the probabilities $p_1, p_2, p_*$. Assuming that the expected effects $(OR_1, OR_2, OR_*)$ have been pre-specified in advance, we obtain estimates of the probabilities under the control $(\hat{p}_1^{(0)}, \hat{p}_2^{(0)}, \hat{p}_*^{(0)})$

and under the treatment group $(\hat{p}_1^{(1)}, \hat{p}_2^{(1)}, \hat{p}_*^{(1)})$. Taking into account the expression (1), we obtained the estimated correlations per arm $\hat{\rho}^{(0)}, \hat{\rho}^{(1)}$

(2) Sample size composite: We reassess the sample size for the composite endpoint by means of the sample size formula in (2.2) in the paper using the estimated event probability $\hat{p}_*^{(0)}$ and the expected effect $OR_*$. Analogously, the sample size for the most relevant component is obtained by using $\hat{p}_1^{(0)}$ and $OR_1$

(3) Compute the decision rule: Based on the estimated sample size, we estimate the decision rule of the ratio of sample sizes, in (3.5) in the paper.

(4) Decision and sample size reassessment: We select the primary endpoint based on the decision rule as explained in the paper and reassess the sample size accordingly.

Note that to allow for different correlations and blinded selection, one has to fix the effect size not only for the components, but also for the composite endpoint. Hence, there is a trade-off by having fewer assumptions but more fixed design parameters.

# 4 Other association measures between binary endpoints

Pearson's correlation is the most common measure to quantify the degree of association between binary endpoints, there are, however, more intuitive alternative measures to define the association between two binary outcomes. In this section, we present the relative overlap and the conditional probability as different ways to measure the association between two binary endpoints.

We relate both measures with the correlation and we rewrite $p_*^{(i)}$ in terms of each of them. In case it is easier to anticipate the association between the components of the composite endpoint using these measures, one can then use them and then the relationship between these measures and the correlation to anticipate the value of the correlation.

## 4.1 Relative overlap

The relative overlap in the $i$-th group of treatment is defined as the conditional probability of observing the two marginal events knowing that at least one of these events has occurred [1]. This measure is evaluated as the ratio between the probability of the intersection, $p_\cap^{(i)}$, and the probability of the composite endpoint, as follows:

$$RO^{(i)} = \frac{p_\cap^{(i)}}{p_*^{(i)}} = \frac{p_\cap^{(i)}}{p_1^{(i)} + p_2^{(i)} - p_\cap^{(i)}} \tag{5}$$

This measure quantifies the ratio of the intersection versus the union of having these two events. The relative overlap takes values between 0 and 1 and is bounded by:

$$RO^{(i)} \in \left[ \max\{0, p_1^{(i)} + p_2^{(i)} - 1\}, \ \ \min\left\{ \frac{p_1^{(i)}}{p_2^{(i)}}, \frac{p_2^{(i)}}{p_1^{(i)}} \right\} \right] \subseteq [0,1] \tag{6}$$

The relative overlap, $RO^{(i)}$, can be expressed by means of the event rates, $p_1^{(i)}$ and $p_2^{(i)}$, and the correlation, $\rho^{(i)}$, as follows:

$$RO^{(i)} = \frac{\rho^{(i)}\sqrt{p_1^{(i)} p_2^{(i)} q_1^{(i)} q_2^{(i)}} + p_1^{(i)} p_2^{(i)}}{1 - q_1^{(i)} q_2^{(i)} - \rho^{(i)}\sqrt{p_1^{(i)} p_2^{(i)} q_1^{(i)} q_2^{(i)}}}$$

Consequently, the probability of the composite endpoint can be rewritten in terms of the marginal parameters and the relative overlap as follows:

$$p_*^{(i)} = \frac{p_1^{(i)} + p_2^{(i)}}{1 + RO^{(i)}}$$

## 4.2 Conditional probability

The conditional probability of observing the two marginal events is the probability of an event occurring given that another event has occurred. Let $P^{(i)}_{X_1|X_2}$ denote the conditional probability of $X_1$ given $X_2$ in group $i$, defined as follows:

$$P^{(i)}_{X_1|X_2} = P(X_{ij1} = 1|X_{ij2} = 1) = \frac{p_\cap^{(i)}}{p_2^{(i)}} \tag{7}$$

This measure quantifies the ratio of the intersection probability over the probability of having had the event $\varepsilon_2$. Note that the conditional probability is not symmetrical with respect to the role of $X_1$ and $X_2$.

The conditional probability takes values between 0 and 1 and is parametrically bounded:

$$P^{(i)}_{X_1|X_2} \in \left[p_1^{(i)}, \min\left\{\frac{p_1^{(i)}}{p_2^{(i)}}, 1\right\}\right] \tag{8}$$

Note that, if $p_1^{(i)} < p_2^{(i)}$, then the bounds are simplified to $\left[p_1^{(i)}, \frac{p_1^{(i)}}{p_2^{(i)}}\right]$; otherwise, $\left[p_1^{(i)}, 1\right]$.

The relationship between the conditional probability and the correlation is given by:

$$P^{(i)}_{X_1|X_2} = \frac{p_1^{(i)} \cdot p_2^{(i)} + \rho^{(i)}\sqrt{p_1^{(i)} p_2^{(i)} q_1^{(i)} q_2^{(i)}}}{p_2^{(i)}} = p_1^{(i)} + \rho^{(i)} \cdot \frac{\sqrt{p_1^{(i)} p_2^{(i)} q_1^{(i)} q_2^{(i)}}}{p_2^{(i)}}$$

The probability of the composite endpoint can be expressed as well in terms of the conditional probabilities and the probabilities of the composite components, as follows:

$$p_*^{(i)} = p_1^{(i)} + p_2^{(i)} - P^{(i)}_{X_1|X_2} \cdot p_2^{(i)}$$

# 5 `eselect` R package

The R package `eselect` implements the calculations for the proposed adaptive design. The flow diagram in Figure 1 in the paper illustrates the algorithm, the required inputs and outputs at each step, and the functions used for its implementation. The R package is available at: https://github.com/MartaBofillRoig/eselect.

The function `eselect` selects between the composite endpoint or the most relevant endpoint as the primary endpoint of the study and recalculates the sample size accordingly, based on data obtained either at an interim analysis or at the end of the trial. The function call is

```
eselect(db, p0_e1, p0_e2, OR1, OR2, alpha = 0.05, beta = 0.2)
```

where `db` is a $2 \times 2$ table with the event counts from the pooled (blinded) sample used to estimate the event probabilities; `p0_e1` and `p0_e2` refer to the event probabilities of the components in the control group assumed in the design stage (that is, $p_1^{(0)}$ and $p_2^{(0)}$); `OR1` and `OR2` are the odds ratios for the components $(OR_1, OR_2)$, and `alpha` and `beta` are the type 1 and 2 errors used to calculate the sample size in respect of the one-sided

tests (2.1) and (2.4) in the paper. The sample size and effect sizes for composite endpoints, necessary for the calculation of the decision rule, are computed using the R-package `CompAREdesign`.

The function `eselectsim` simulates trials with adaptive endpoint selection and sample size reassessment for composite binary endpoints with two components. The function uses the algorithm implemented in `eselect` to select the primary endpoint and recalculate the sample size. The function call is

```
eselectsim(ss_arm, p0_e1, p0_e2, OR1, OR2, p0_ce, p_init = 1,
           H0_e1 = FALSE, H0_e2 = FALSE, SS_r = TRUE, alpha = 0.05, beta = 0.2)
```

where `p0_e1`, `p0_e2`, `OR1` and `OR2`, `alpha` and `beta` are the same arguments used in `eselect`; `ss_arm` is the sample size per arm (assuming equal randomization); `p_init` is the percentage of sample size used for estimating the probabilities in the control and the correlation and selecting the design; `H0_e1` and `H0_e2` indicate the simulations are performed under the null hypothesis for the composite endpoints, and `SS_r` indicates whether the sample size is reassessed after the endpoint selection.

Other functions for designs using unblinded data instead of blinded data, as is explained in this supplementary material (see Section 2) and discussed in the simulation study in the paper, are available in the R package.

# 6 Example with R: Target-vessel revascularization in cardiology trials

In patients with coronary artery disease, the composite binary endpoint $\varepsilon_*$ of ischemia-driven target-vessel revascularization ($\varepsilon_1$) and death from cardiac causes or myocardial infarction ($\varepsilon_2$) has been considered for evaluating the efficacy and safety of different stents in cardiology trials. As an example, TAXUS-IV was a randomized controlled clinical trial (RCT) to investigate the safety and efficacy of a placlitaxel-eluting stent in a patient population with coronary artery disease [2]. The primary endpoint was ischemia-driven target-vessel revascularization, considered the most relevant of the composite components, while the composite endpoint was considered a secondary endpoint. Subsequently, TAXUS-V trial was conducted to evaluate the efficacy of such stents in a patient population with more complex lesions than the one studied in the previous trial. In TAXUS-V trial [3], the same endpoints were considered. TAXUS-V was also a RCT with a total of n=1145 patients allocated 1:1 to the two treatment groups. The final result was that the primary endpoint ($\varepsilon_1$) was statistically significant (p=0.02) with observed rates of 0.173 and 0.121 for the two groups, respectively.

In what follows, we exemplify the use of the proposed adaptive design and the R package `eselect` using blinded data from TAXUS-V, according only to results presented in Stone et al. [2].

## 6.1 PART A: Re-analysis of TAXUS-V with adaptive endpoint selection based on blinded data with different odd ratios for the components

Here, we illustrate how the proposed adaptive design could have been used for adaptive selection of the primary endpoint before conducting the final analysis and unblinding of the data. To do so, we will use the R packages `CompAREdesign` and `eselect`, which one can install through CRAN and GitHub:

```
library(CompAREdesign)
devtools::install_github("MartaBofillRoig/eselect")
```

For illustrative purposes, we consider the total number of patients included in primary analysis as the initial sample size, $\tilde{n} = 1145$, where the endpoint selection is performed. For the selection of the endpoint assumptions have to be made according to the expected event probabilities in the control group and respective effect sizes in terms of odd ratios. The values were fixed to `p0_e1 = 0.18` and `p0_e2 = 0.050`, for the event probabilities of $\varepsilon_1$ and $\varepsilon_2$, and consider expected effect sizes for the components of `OR1=0.70` and `OR2=0.90`.

We used the blinded data collected at the end of the study in TAXUS-V for selecting the primary endpoint according the proposed adaptive design in Section 3 of the paper. In particular, we used the data in a blinded way to estimate the event probabilities in the pooled sample (see Section 2 of this supplementary material) and obtain estimates of the event probabilities in the control arm and correlation to compute the decision rule, as explained in Sections 3.1 and 3.2 of the paper.

The following $2 \times 2$ table (Table 1) summarizes the blinded data obtained at the end of TAXUS-V trial regarding the components of the composite endpoint, $\varepsilon_1$ and $\varepsilon_2$. Based on that data, we can use the function

| | Patients with ischemia-driven target-vessel revascularization $\varepsilon_1$ | Patients without $\varepsilon_1$ |
|---|---|---|
| Patients with cardiac death or myocardial infarction $\varepsilon_2$ | 33 | 31 |
| Patients without $\varepsilon_2$ | 135 | 945 |

Table 1: Blinded data at the end of the trial for TAXUS-V (based on Table 3 in Stone et al. [2]).

`eselect` to implement the adaptive selection as follows

```
> eselect(db=data,p0_e1=0.18,OR1=0.70,p0_e2=0.05,OR2=0.9,alpha=0.05,beta=0.2)
$SampleSize
[1] 1582.689
$Decision
[1] 0
```

where `data` refers to data in Table 1. The function returns the decision (Decision = 1, meaning the selected endpoint is the composite endpoint; and Decision = 0, meaning the selected endpoint is the relevant endpoint) and the sample size needed to test the primary hypothesis according to the decision to achieve 80% at $\alpha = 0.05$ significance level. Hence, in this case the decision is to keep the relevant endpoint as the primary endpoint of the trial. If an adaptive sample size reassessment was part of the initial design, then the sample size would have to be increased to achieve the targeted power. Otherwise, if only the adaptive selection of the endpoint was part of the design, the final analysis could then be performed with unblinded data. For the latter case the results would not have changed compared with TAXUS-V, as the same primary endpoint was selected.

## 6.2 Part B: Endpoint selection assuming different effect sizes and correlations using TAXUS-V

By means of the function `eselectsim`, we simulate trials to evaluate the adaptive selection depending on different scenarios. As before we assume that the selection should be made at the end of the trial with a total sample of $n = 1145$ as observed in TAXUS-V. For the blinded endpoint selection, we consider the event probabilities in the control arm of the components assumed at the planning stage as note above and same expected effect size for $\varepsilon_1$, and suppose different values for the event probability of the composite endpoint and the expected effect size of $\varepsilon_2$.

In order to compute plausible values of the probability rate of the composite endpoint based on the event probabilities of the components according to several values of the correlation, we can use the function `prob_cbe` as follows

```
> (p0_ce = prob_cbe(p_e1=0.18, p_e2=0.05, rho=c(0,0.1,0.2,0.3,0.4,0.5)))
[1] 0.2210000 0.2126268 0.2042537 0.1958805 0.1875073 0.1791341
```

which give the event probability of the composite endpoint for correlation values of $\rho = 0, 0.1, 0.2, 0.3, 0.4, 0.5$.

Using the obtained values for the event probability of the composite endpoint, then we use `eselectsim` to evaluate which is the selected endpoint in the adaptive design. This function returns the decision (Decision =

1, meaning the selected endpoint is the composite endpoint; and Decision = 0, meaning the selected endpoint is the relevant endpoint) and the statistic to test the primary hypothesis according to the decision.

If the effect size of $\varepsilon_2$ is expected to be equal to $OR_2 = 0.80$, then obtain

```
> eselectsim(ss_arm=n/2, p0_e1=0.18, OR1=OR1, p0_e2=0.05, OR2=0.80, p0_ce=p0_ce[1],
p_init = 1, SS_r=F, alpha = 0.05, beta = 0.2)
$Test
[1] -1.265482
$Decision
[1] 1
> eselectsim(ss_arm=n/2, p0_e1=0.18, OR1=OR1, p0_e2=0.05, OR2=0.80, p0_ce=p0_ce[3],
p_init = 1, SS_r=F, alpha = 0.05, beta = 0.2)
$Test
[1] -2.78344
$Decision
[1] 1
> eselectsim(ss_arm=n/2, p0_e1=0.18, OR1=OR1, p0_e2=0.05, OR2=0.80, p0_ce=p0_ce[4],
p_init = 1, SS_r=F, alpha = 0.05, beta = 0.2)
$Test
[1] -2.453637
$Decision
[1] 0
```

If we assume the probability rate of the composite endpoint is the one obtained assuming correlations smaller than 0.3, then the decision is to use the composite endpoint as primary endpoint, while if the correlation is assumed to be larger than 0.4, then the most relevant endpoint is selected.

If, however, the expected effect size of $\varepsilon_2$ equals to $OR_2 = 0.90$, then the most relevant endpoint is selected regardless the value of the correlation between components

```
> eselectsim(ss_arm=n/2, p0_e1=0.18, OR1=OR1, p0_e2=0.05, OR2=0.90, p0_ce=p0_ce[1],
p_init = 1, SS_r=F, alpha = 0.05, beta = 0.2)
$Test
[1] -2.472174
$Decision
[1] 0
> eselectsim(ss_arm=n/2, p0_e1=0.18, OR1=OR1, p0_e2=0.05, OR2=0.90, p0_ce=p0_ce[3],
p_init = 1, SS_r=F, alpha = 0.05, beta = 0.2)
$Test
[1] -2.161327
$Decision
[1] 0
> eselectsim(ss_arm=n/2, p0_e1=0.18, OR1=OR1, p0_e2=0.05, OR2=0.90, p0_ce=p0_ce[4],
p_init = 1, SS_r=F, alpha = 0.05, beta = 0.2)
$Test
[1] -2.252063
$Decision
[1] 0
```

# 7 Additional results simulation study

In this section, we add further results obtained in the simulation study. In 7.1, we summarize the results obtained when using the proposed adaptive design but with unblinded data. In 7.2 and 7.3, we depict how

the adaptive design behaves in terms of the power for all the scenarios considered in the simulation study as compared to the composite and relevant designs and with respect to the correlation between the composite components. Note that each column corresponds to a specific parameter constellation for $(p_1^{(0)}, p_2^{(0)}, OR_1, OR_2)$. Tables on the bottom shows the value of the decision rule computed using the parameters' values used for the simulation and the percentage of cases in which the composite endpoint is selected as the primary endpoint (denoted by %CE) .

## 7.1 Results for designs using unblinded data

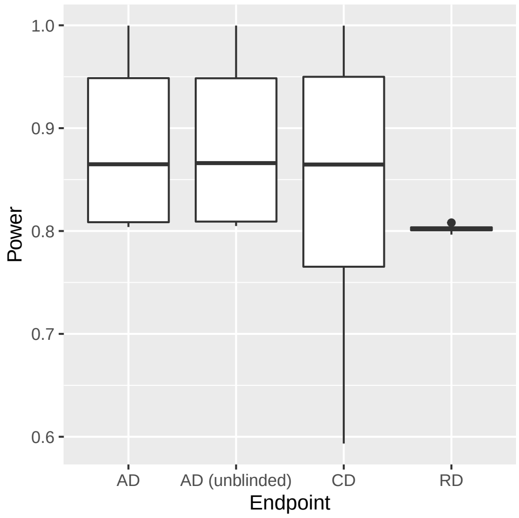


(a) Without sample size reassessment

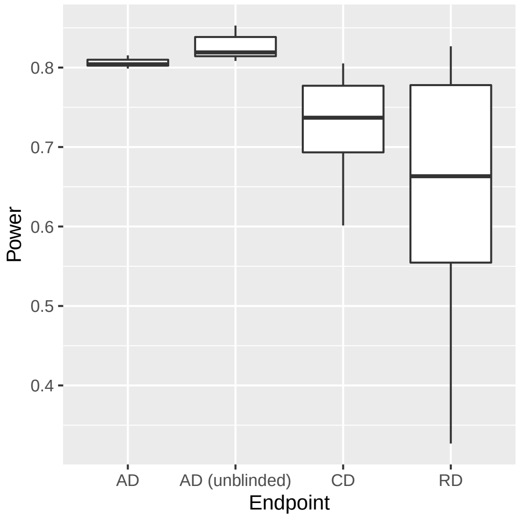


(b) With sample size reassessment

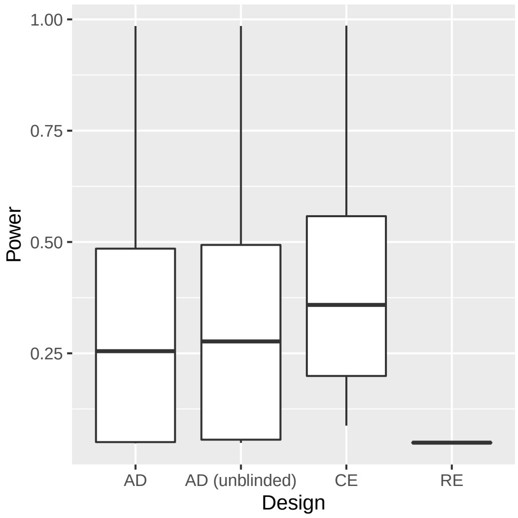


(c) Non-effect on the relevant endpoint

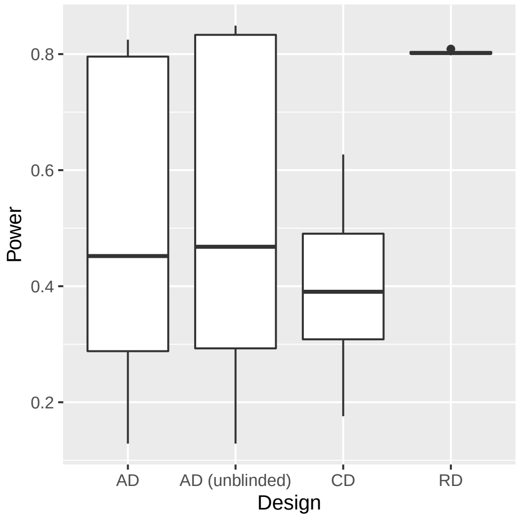


(d) Non-effect on the second composite component

Figure 2: Power under composite design (CD), relevant design (RD) and adaptive design (AD). Case (a) refers to designs with selection at the end of the trial without sample size recalculation for which the initial sample size was computed to have 0.80 power to detect effects on the relevant endpoint. Case (b) refers to those designs with selection at the interim analysis and with sample size recalculation for which the sample size was computed the have 0.80 power to detect effects on the composite endpoint assuming zero correlation between the components. Cases (c) and (d) refer to situations where one of the components had no effect and to designs with interim analysis and for which the initial sample size was computed to have 0.80 power to detect effects on the relevant endpoint.

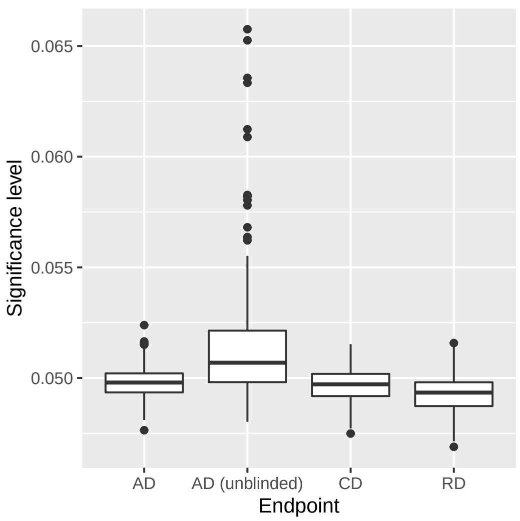


Figure 3: Observed type 1 error rates based on 242 null scenarios (see Table 2 in the main paper) under composite design (CD), relevant design (RD) and adaptive design (AD).

## 7.2 Additional results with sample size assessment

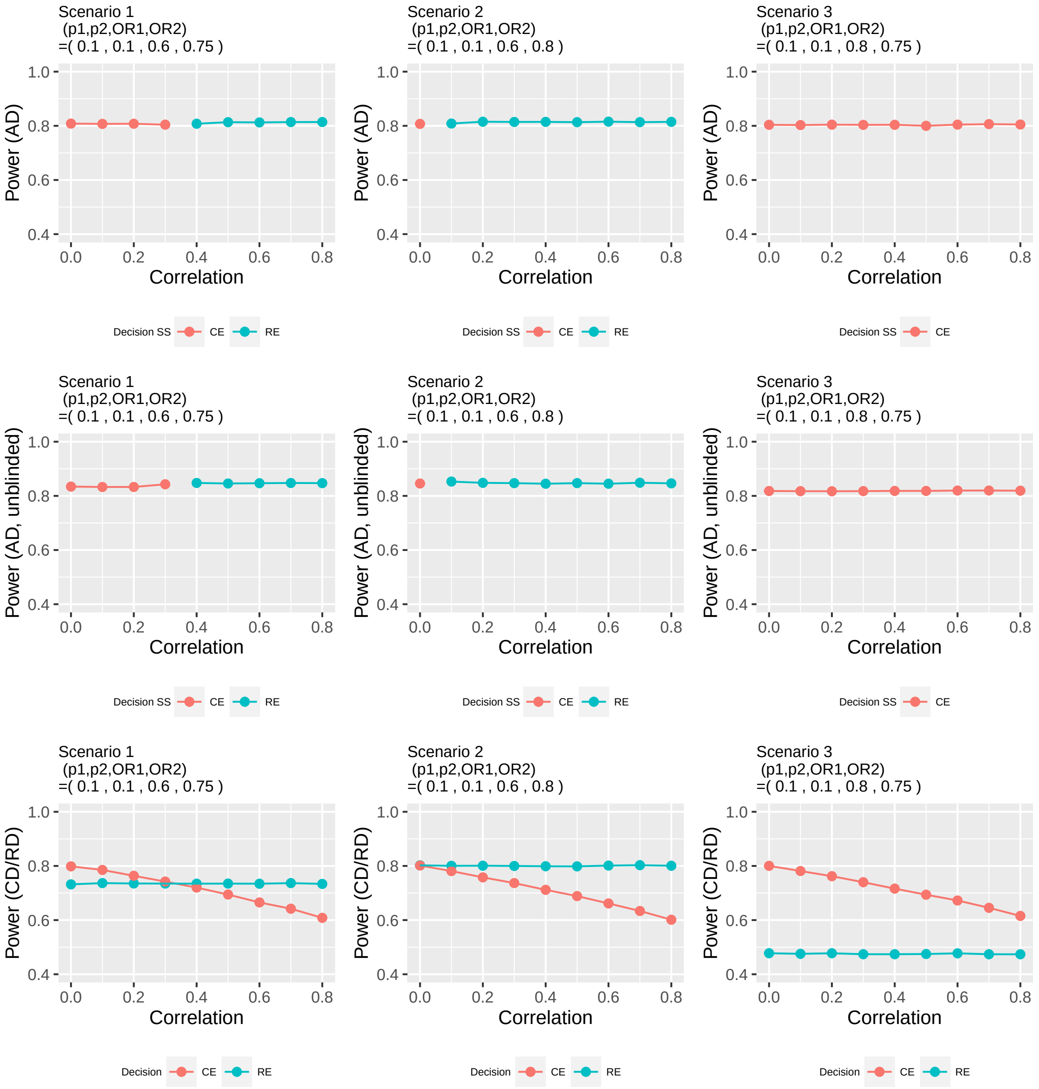


| Correlation | Decision rule | % CE |
|---|---|---|
| 0 | 1.21 | 100 |
| 0.1 | 1.14 | 99.93 |
| 0.2 | 1.08 | 95.44 |
| 0.3 | 1.02 | 64.76 |
| 0.4 | 0.96 | 20.44 |
| 0.5 | 0.9 | 2.62 |
| 0.6 | 0.84 | 0.14 |
| 0.7 | 0.78 | 0 |
| 0.8 | 0.72 | 0 |

| Correlation | Decision rule | % CE |
|---|---|---|
| 0 | 1.01 | 71.07 |
| 0.1 | 0.96 | 11.78 |
| 0.2 | 0.9 | 0.69 |
| 0.3 | 0.85 | 0.01 |
| 0.4 | 0.8 | 0 |
| 0.5 | 0.75 | 0 |
| 0.6 | 0.7 | 0 |
| 0.7 | 0.65 | 0 |
| 0.8 | 0.6 | 0 |

| Correlation | Decision rule | % CE |
|---|---|---|
| 0 | 2.45 | 100 |
| 0.1 | 2.33 | 100 |
| 0.2 | 2.2 | 100 |
| 0.3 | 2.08 | 100 |
| 0.4 | 1.96 | 100 |
| 0.5 | 1.84 | 100 |
| 0.6 | 1.72 | 100 |
| 0.7 | 1.61 | 100 |
| 0.8 | 1.5 | 100 |

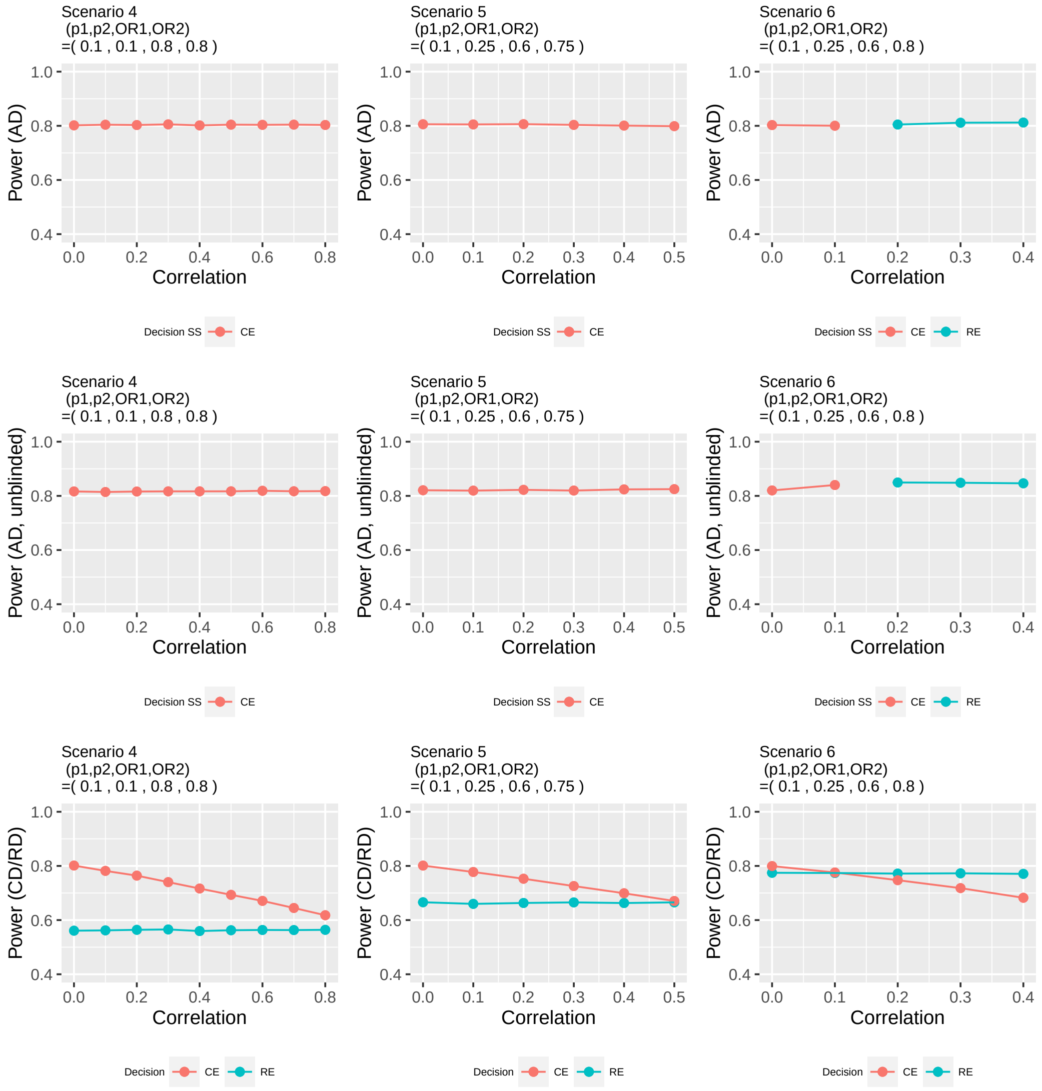


| Correlation | Decision rule | % CE |
|---|---|---|
| 0 | 1.91 | 100 |
| 0.1 | 1.81 | 100 |
| 0.2 | 1.71 | 100 |
| 0.3 | 1.62 | 100 |
| 0.4 | 1.52 | 100 |
| 0.5 | 1.43 | 100 |
| 0.6 | 1.34 | 100 |
| 0.7 | 1.25 | 100 |
| 0.8 | 1.17 | 100 |

| Correlation | Decision rule | % CE |
|---|---|---|
| 0 | 1.48 | 100 |
| 0.1 | 1.38 | 100 |
| 0.2 | 1.29 | 99.99 |
| 0.3 | 1.2 | 99.22 |
| 0.4 | 1.12 | 90.94 |
| 0.5 | 1.03 | 68.47 |

| Correlation | Decision rule | % CE |
|---|---|---|
| 0 | 1.1 | 97.87 |
| 0.1 | 1.02 | 66.8 |
| 0.2 | 0.95 | 22.14 |
| 0.3 | 0.87 | 4.1 |
| 0.4 | 0.8 | 0.43 |

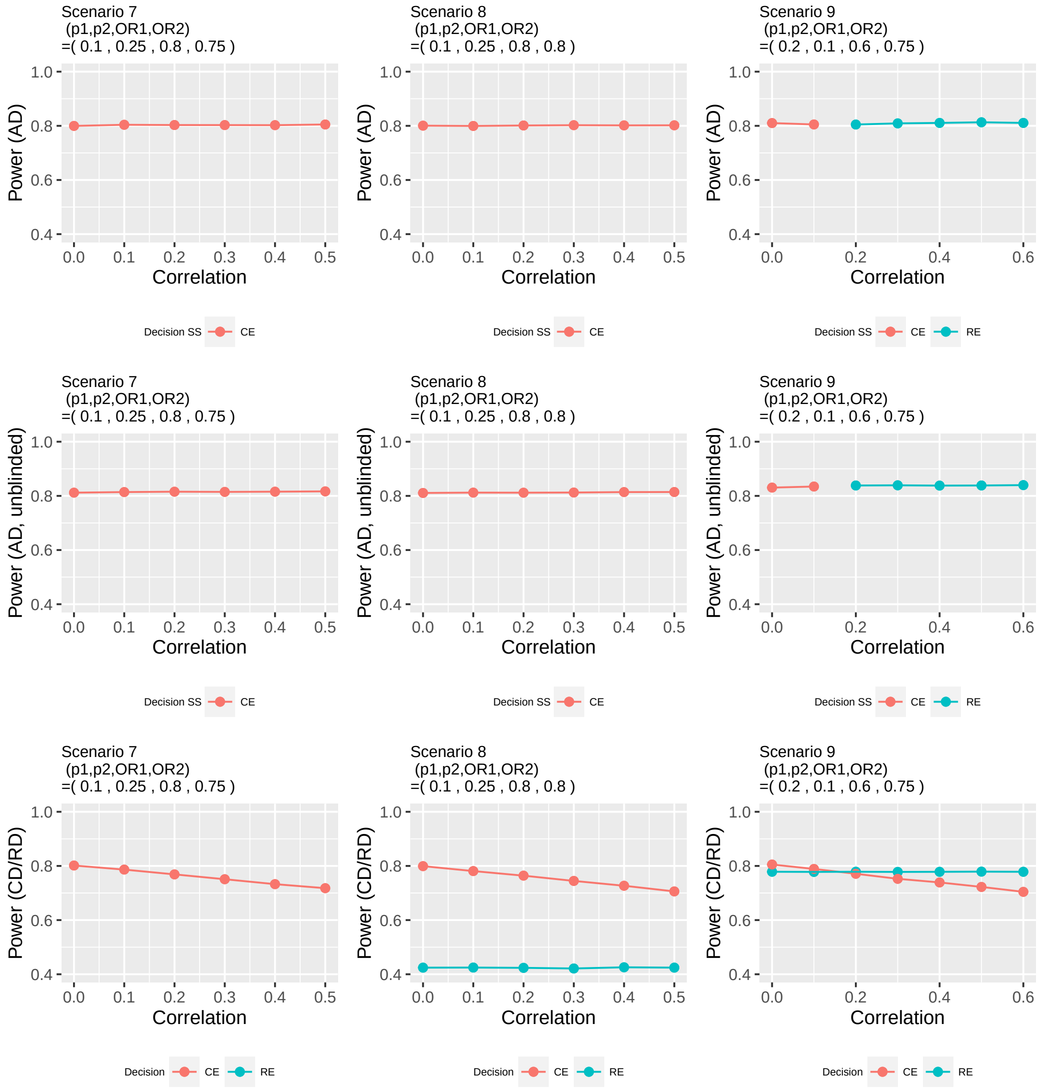


| Correlation | Decision rule | % CE |
|---|---|---|
| 0 | 4.19 | 100 |
| 0.1 | 4.01 | 100 |
| 0.2 | 3.83 | 100 |
| 0.3 | 3.66 | 100 |
| 0.4 | 3.49 | 100 |
| 0.5 | 3.33 | 100 |

| Correlation | Decision rule | % CE |
|---|---|---|
| 0 | 2.92 | 100 |
| 0.1 | 2.78 | 100 |
| 0.2 | 2.64 | 100 |
| 0.3 | 2.5 | 100 |
| 0.4 | 2.37 | 100 |
| 0.5 | 2.25 | 100 |

| Correlation | Decision rule | % CE |
|---|---|---|
| 0 | 1.08 | 99.58 |
| 0.1 | 1.03 | 84.37 |
| 0.2 | 0.99 | 35.33 |
| 0.3 | 0.94 | 4.8 |
| 0.4 | 0.9 | 0.18 |
| 0.5 | 0.86 | 0 |
| 0.6 | 0.82 | 0 |

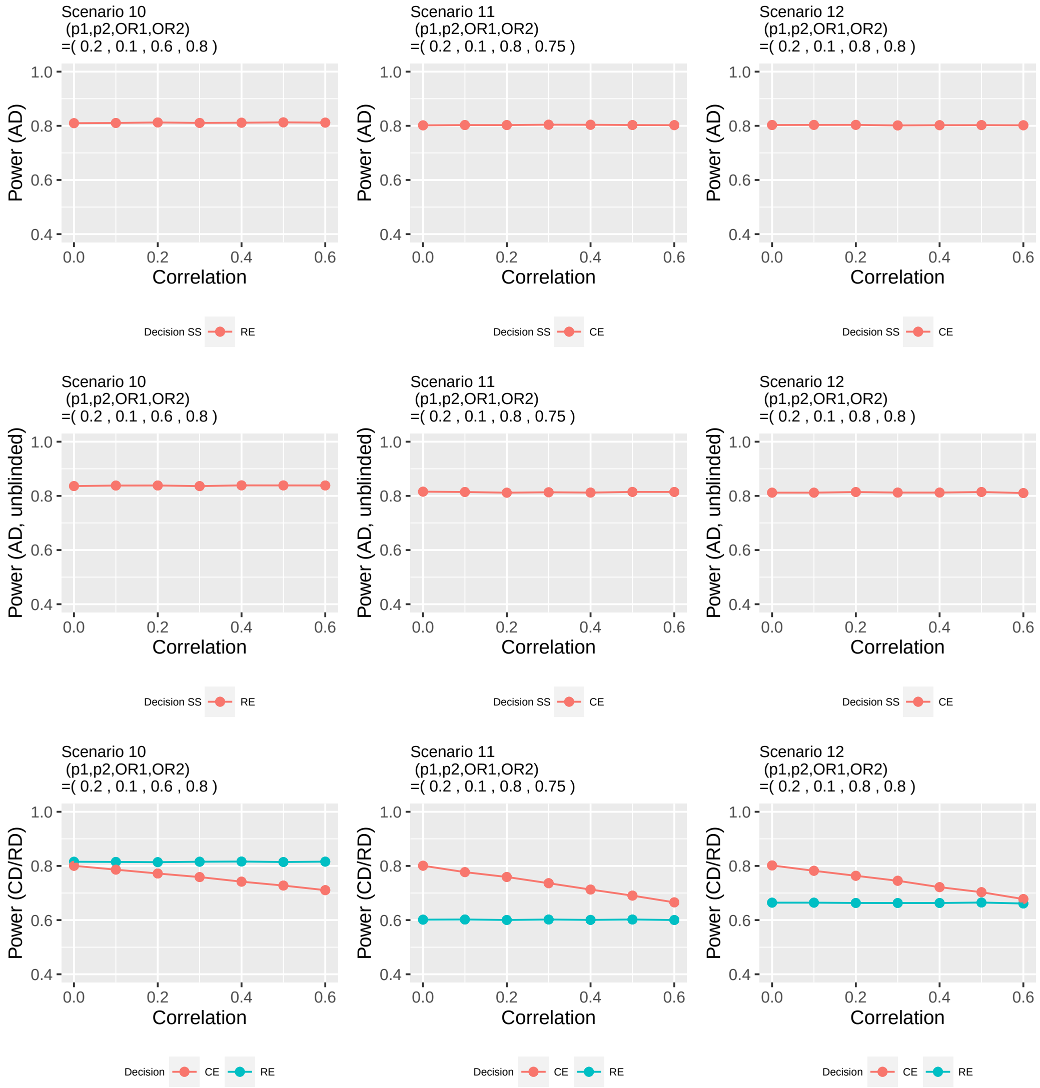


| Correlation | Decision rule | % CE |
|---|---|---|
| 0 | 0.97 | 6.98 |
| 0.1 | 0.93 | 0.18 |
| 0.2 | 0.89 | 0 |
| 0.3 | 0.86 | 0 |
| 0.4 | 0.82 | 0 |
| 0.5 | 0.79 | 0 |
| 0.6 | 0.75 | 0 |

| Correlation | Decision rule | % CE |
|---|---|---|
| 0 | 1.72 | 100 |
| 0.1 | 1.62 | 100 |
| 0.2 | 1.53 | 100 |
| 0.3 | 1.44 | 100 |
| 0.4 | 1.35 | 100 |
| 0.5 | 1.27 | 100 |
| 0.6 | 1.19 | 100 |

| Correlation | Decision rule | % CE |
|---|---|---|
| 0 | 1.45 | 100 |
| 0.1 | 1.37 | 100 |
| 0.2 | 1.3 | 100 |
| 0.3 | 1.24 | 100 |
| 0.4 | 1.17 | 100 |
| 0.5 | 1.1 | 100 |
| 0.6 | 1.04 | 99.99 |

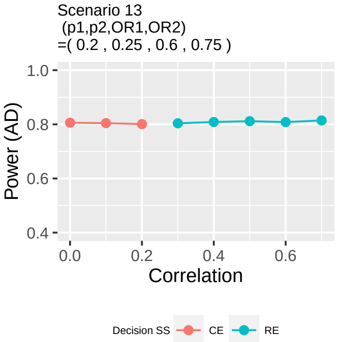


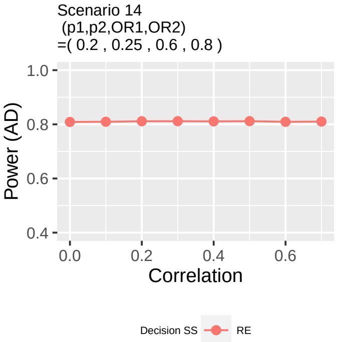


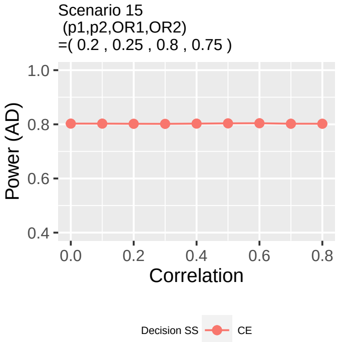


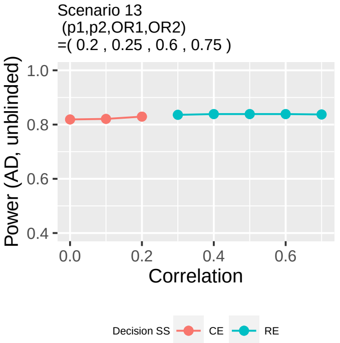


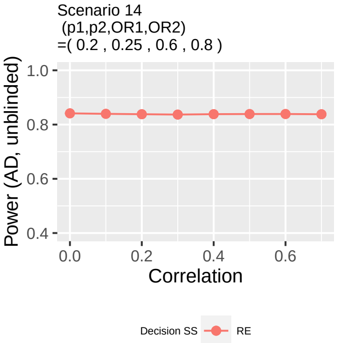


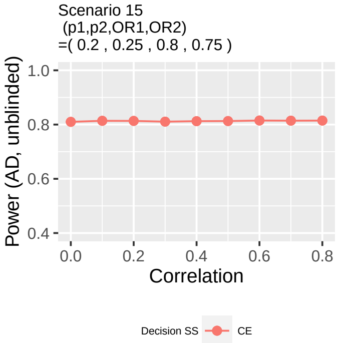


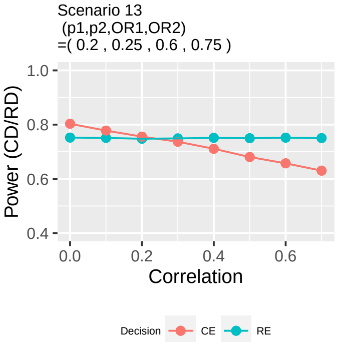


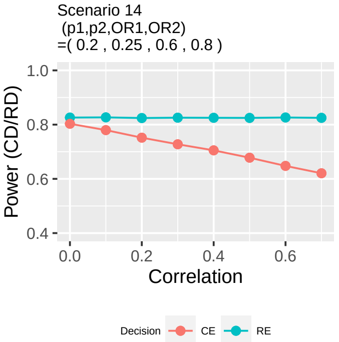


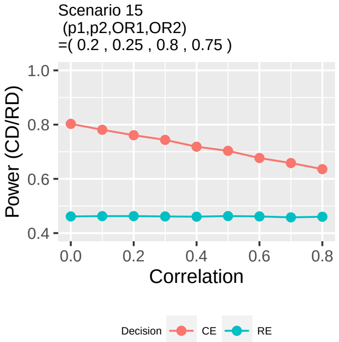


| Correlation | Decision rule | % CE |
|---|---|---|
| 0 | 1.15 | 99.94 |
| 0.1 | 1.08 | 95.49 |
| 0.2 | 1.02 | 64.75 |
| 0.3 | 0.96 | 21.29 |
| 0.4 | 0.9 | 3.09 |
| 0.5 | 0.84 | 0.21 |
| 0.6 | 0.79 | 0.01 |
| 0.7 | 0.73 | 0 |

| Correlation | Decision rule | % CE |
|---|---|---|
| 0 | 0.94 | 4.71 |
| 0.1 | 0.88 | 0.15 |
| 0.2 | 0.82 | 0 |
| 0.3 | 0.77 | 0 |
| 0.4 | 0.72 | 0 |
| 0.5 | 0.67 | 0 |
| 0.6 | 0.63 | 0 |
| 0.7 | 0.58 | 0 |

| Correlation | Decision rule | % CE |
|---|---|---|
| 0 | 2.58 | 100 |
| 0.1 | 2.44 | 100 |
| 0.2 | 2.32 | 100 |
| 0.3 | 2.19 | 100 |
| 0.4 | 2.08 | 100 |
| 0.5 | 1.97 | 100 |
| 0.6 | 1.86 | 100 |
| 0.7 | 1.76 | 100 |
| 0.8 | 1.67 | 100 |

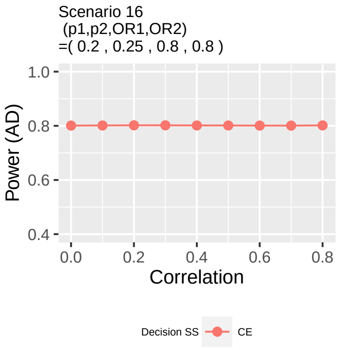


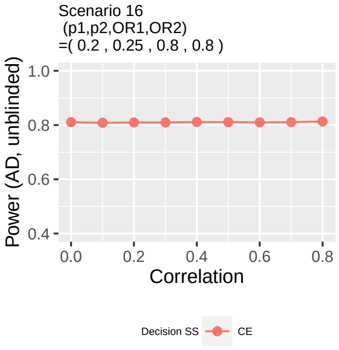


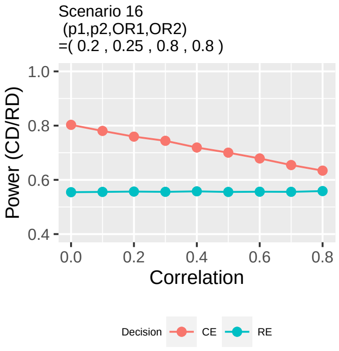


| Correlation | Decision rule | % CE |
|---|---|---|
| 0 | 1.94 | 100 |
| 0.1 | 1.83 | 100 |
| 0.2 | 1.73 | 100 |
| 0.3 | 1.64 | 100 |
| 0.4 | 1.55 | 100 |
| 0.5 | 1.46 | 100 |
| 0.6 | 1.38 | 100 |
| 0.7 | 1.3 | 100 |
| 0.8 | 1.23 | 100 |

## 7.3 Additional results without sample size assessment

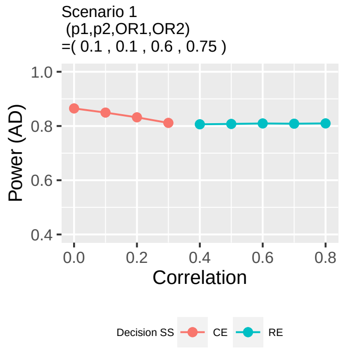


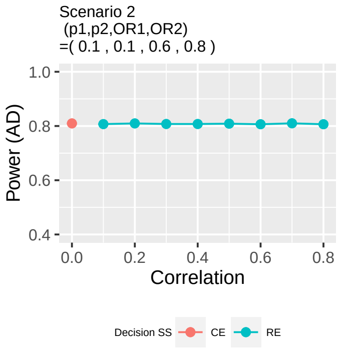


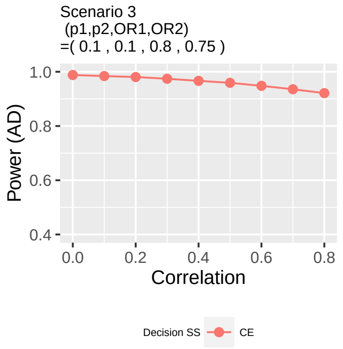


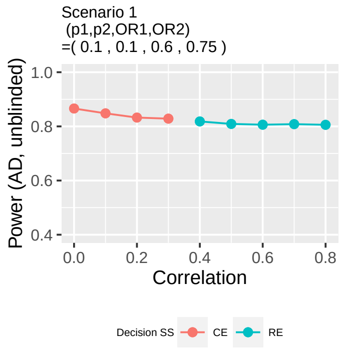


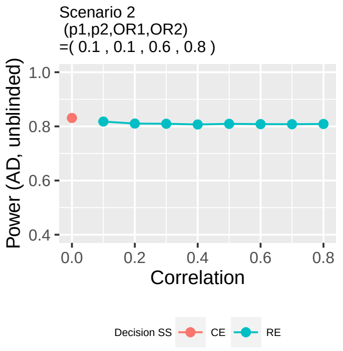


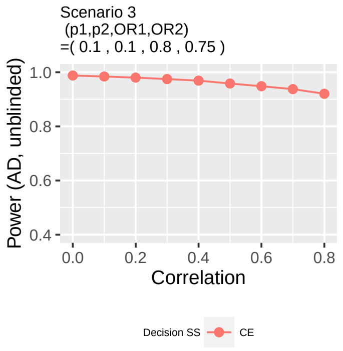


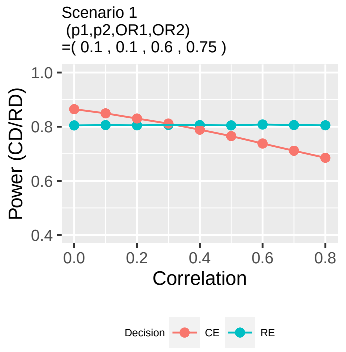


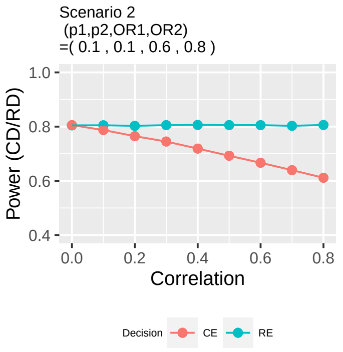


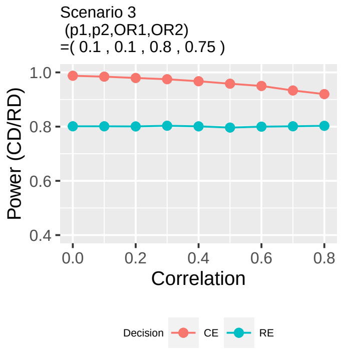


| Correlation | Decision rule | % CE |
|---|---|---|
| 0 | 1.21 | 100 |
| 0.1 | 1.14 | 100 |
| 0.2 | 1.08 | 99.57 |
| 0.3 | 1.02 | 71.96 |
| 0.4 | 0.96 | 9.83 |
| 0.5 | 0.9 | 0.11 |
| 0.6 | 0.84 | 0 |
| 0.7 | 0.78 | 0 |
| 0.8 | 0.72 | 0 |

| Correlation | Decision rule | % CE |
|---|---|---|
| 0 | 1.01 | 77.39 |
| 0.1 | 0.96 | 4.11 |
| 0.2 | 0.9 | 0.02 |
| 0.3 | 0.85 | 0 |
| 0.4 | 0.8 | 0 |
| 0.5 | 0.75 | 0 |
| 0.6 | 0.7 | 0 |
| 0.7 | 0.65 | 0 |
| 0.8 | 0.6 | 0 |

| Correlation | Decision rule | % CE |
|---|---|---|
| 0 | 2.45 | 100 |
| 0.1 | 2.33 | 100 |
| 0.2 | 2.2 | 100 |
| 0.3 | 2.08 | 100 |
| 0.4 | 1.96 | 100 |
| 0.5 | 1.84 | 100 |
| 0.6 | 1.72 | 100 |
| 0.7 | 1.61 | 100 |
| 0.8 | 1.5 | 100 |

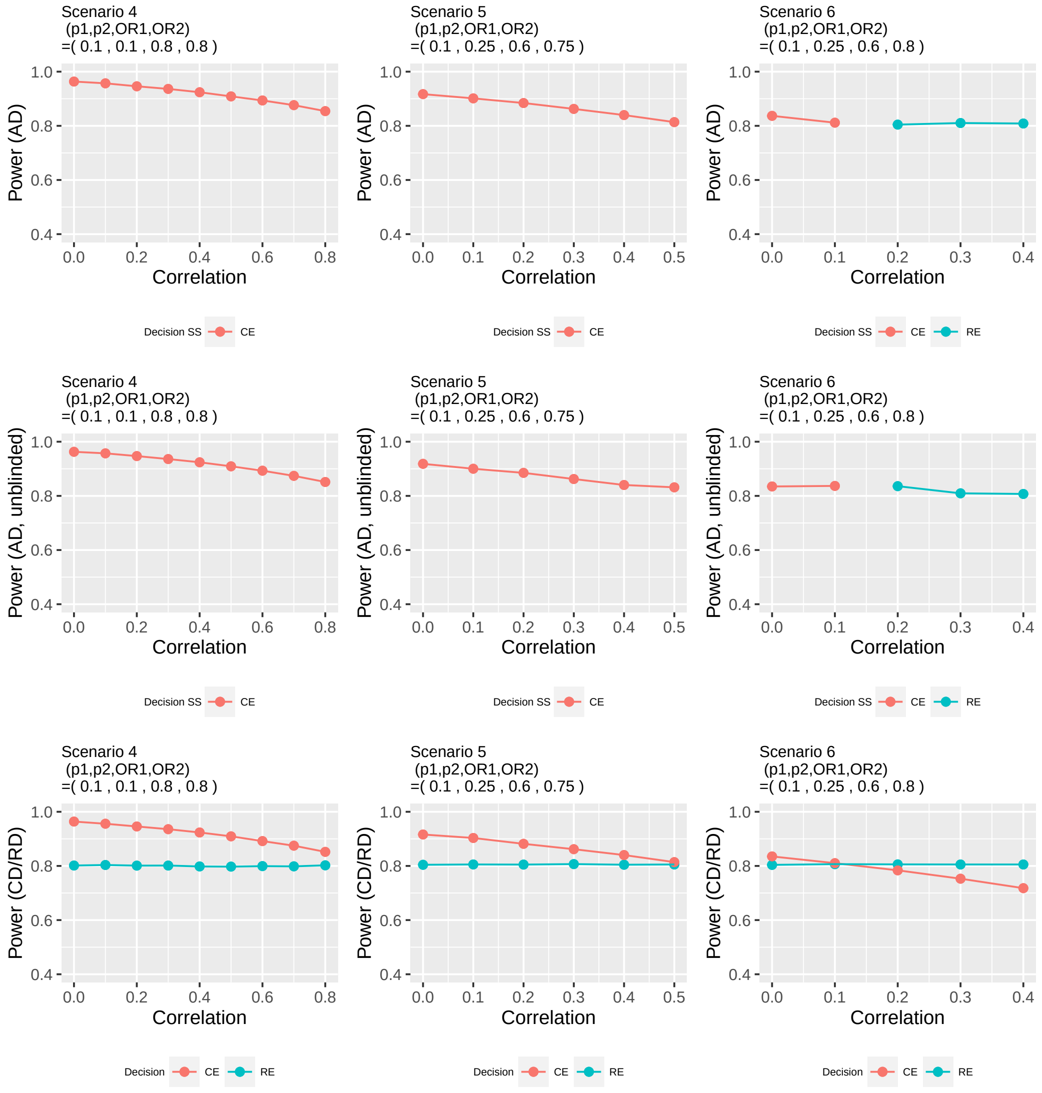


| Correlation | Decision rule | % CE |
|---|---|---|
| 0 | 1.91 | 100 |
| 0.1 | 1.81 | 100 |
| 0.2 | 1.71 | 100 |
| 0.3 | 1.62 | 100 |
| 0.4 | 1.52 | 100 |
| 0.5 | 1.43 | 100 |
| 0.6 | 1.34 | 100 |
| 0.7 | 1.25 | 100 |
| 0.8 | 1.17 | 100 |

| Correlation | Decision rule | % CE |
|---|---|---|
| 0 | 1.48 | 100 |
| 0.1 | 1.38 | 100 |
| 0.2 | 1.29 | 100 |
| 0.3 | 1.2 | 100 |
| 0.4 | 1.12 | 98.95 |
| 0.5 | 1.03 | 77.42 |

| Correlation | Decision rule | % CE |
|---|---|---|
| 0 | 1.1 | 99.86 |
| 0.1 | 1.02 | 73.14 |
| 0.2 | 0.95 | 12.41 |
| 0.3 | 0.87 | 0.45 |
| 0.4 | 0.8 | 0 |

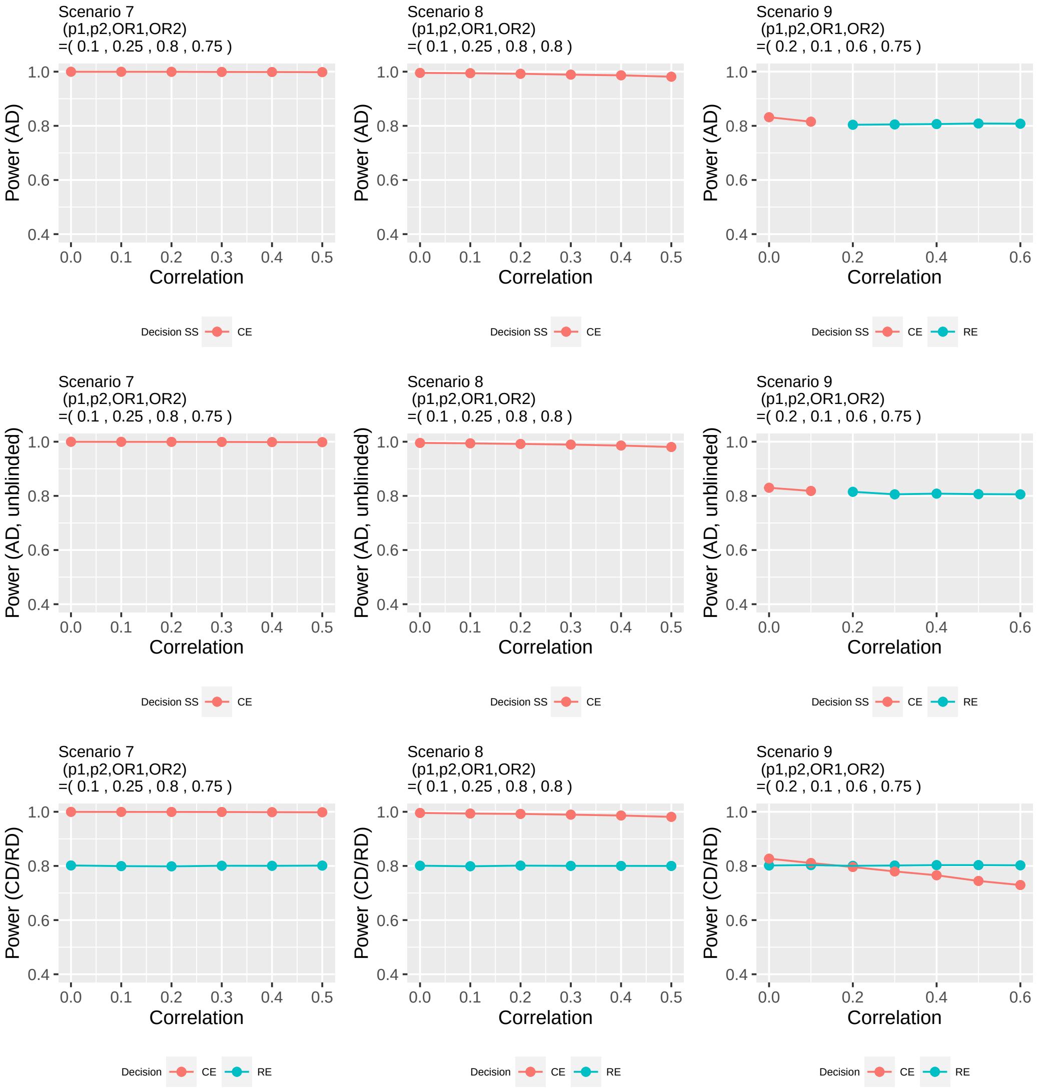


| Correlation | Decision rule | % CE |
|---|---|---|
| 0 | 4.19 | 100 |
| 0.1 | 4.01 | 100 |
| 0.2 | 3.83 | 100 |
| 0.3 | 3.66 | 100 |
| 0.4 | 3.49 | 100 |
| 0.5 | 3.33 | 100 |

| Correlation | Decision rule | % CE |
|---|---|---|
| 0 | 2.92 | 100 |
| 0.1 | 2.78 | 100 |
| 0.2 | 2.64 | 100 |
| 0.3 | 2.5 | 100 |
| 0.4 | 2.37 | 100 |
| 0.5 | 2.25 | 100 |

| Correlation | Decision rule | % CE |
|---|---|---|
| 0 | 1.08 | 100 |
| 0.1 | 1.03 | 92.88 |
| 0.2 | 0.99 | 28.28 |
| 0.3 | 0.94 | 0.69 |
| 0.4 | 0.9 | 0 |
| 0.5 | 0.86 | 0 |
| 0.6 | 0.82 | 0 |

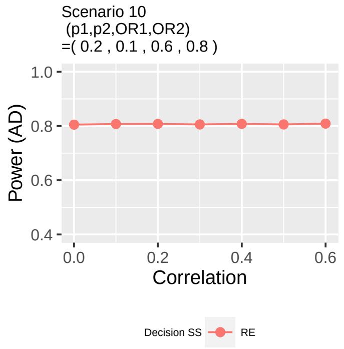


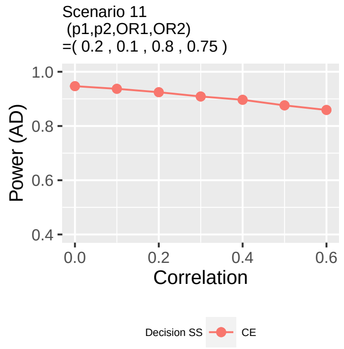


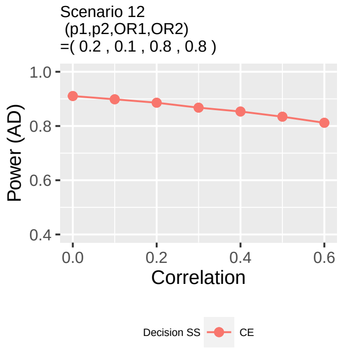


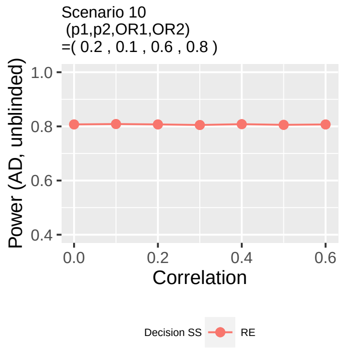


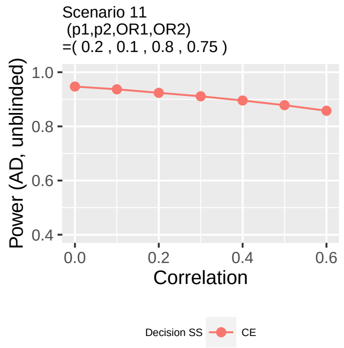


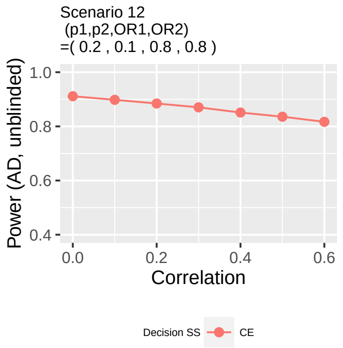


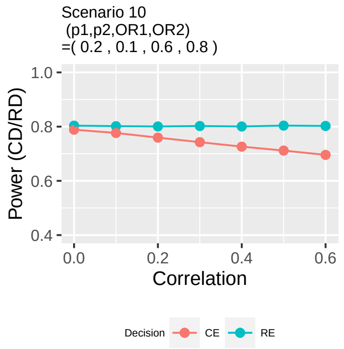


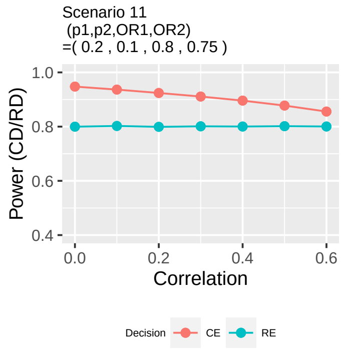


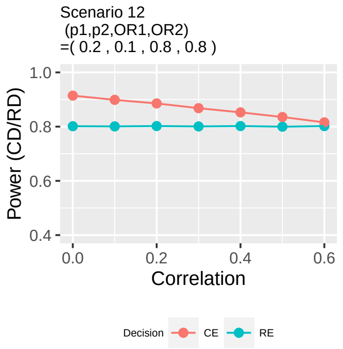


| Correlation | Decision rule | % CE |
|---|---|---|
| 0 | 0.97 | 1.66 |
| 0.1 | 0.93 | 0 |
| 0.2 | 0.89 | 0 |
| 0.3 | 0.86 | 0 |
| 0.4 | 0.82 | 0 |
| 0.5 | 0.79 | 0 |
| 0.6 | 0.75 | 0 |

| Correlation | Decision rule | % CE |
|---|---|---|
| 0 | 1.72 | 100 |
| 0.1 | 1.62 | 100 |
| 0.2 | 1.53 | 100 |
| 0.3 | 1.44 | 100 |
| 0.4 | 1.35 | 100 |
| 0.5 | 1.27 | 100 |
| 0.6 | 1.19 | 100 |

| Correlation | Decision rule | % CE |
|---|---|---|
| 0 | 1.45 | 100 |
| 0.1 | 1.37 | 100 |
| 0.2 | 1.3 | 100 |
| 0.3 | 1.24 | 100 |
| 0.4 | 1.17 | 100 |
| 0.5 | 1.1 | 100 |
| 0.6 | 1.04 | 100 |

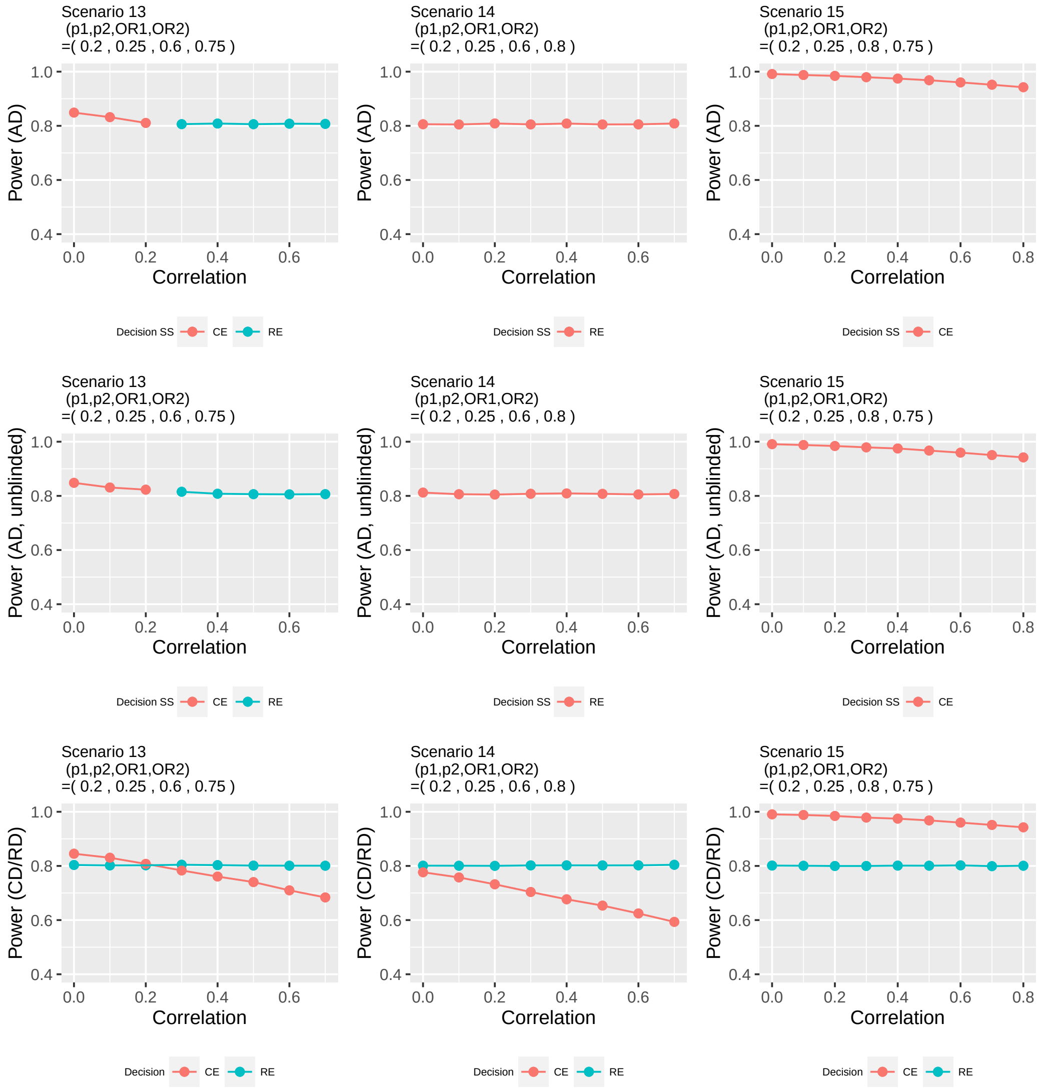


| Correlation | Decision rule | % CE |
|---|---|---|
| 0 | 1.15 | 100 |
| 0.1 | 1.08 | 99.54 |
| 0.2 | 1.02 | 71.46 |
| 0.3 | 0.96 | 11.25 |
| 0.4 | 0.9 | 0.23 |
| 0.5 | 0.84 | 0 |
| 0.6 | 0.79 | 0 |
| 0.7 | 0.73 | 0 |

| Correlation | Decision rule | % CE |
|---|---|---|
| 0 | 0.94 | 0.96 |
| 0.1 | 0.88 | 0 |
| 0.2 | 0.82 | 0 |
| 0.3 | 0.77 | 0 |
| 0.4 | 0.72 | 0 |
| 0.5 | 0.67 | 0 |
| 0.6 | 0.63 | 0 |
| 0.7 | 0.58 | 0 |

| Correlation | Decision rule | % CE |
|---|---|---|
| 0 | 2.58 | 100 |
| 0.1 | 2.44 | 100 |
| 0.2 | 2.32 | 100 |
| 0.3 | 2.19 | 100 |
| 0.4 | 2.08 | 100 |
| 0.5 | 1.97 | 100 |
| 0.6 | 1.86 | 100 |
| 0.7 | 1.76 | 100 |
| 0.8 | 1.67 | 100 |

Scenario 16
(p1,p2,OR1,OR2)
=( 0.2 , 0.25 , 0.8 , 0.8 )

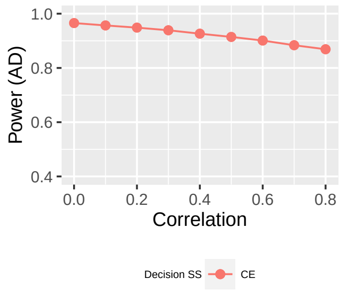


Scenario 16
(p1,p2,OR1,OR2)
=( 0.2 , 0.25 , 0.8 , 0.8 )

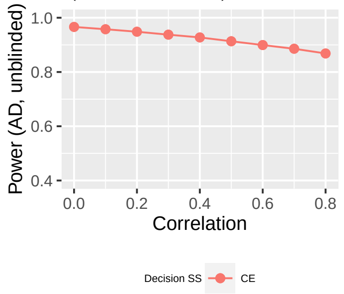


Scenario 16
(p1,p2,OR1,OR2)
=( 0.2 , 0.25 , 0.8 , 0.8 )

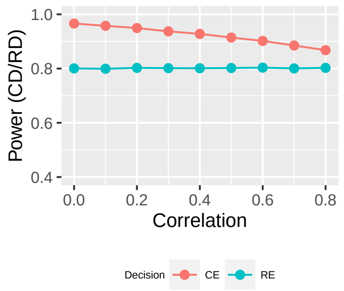


| Correlation | Decision rule | % CE |
|---|---|---|
| 0 | 1.94 | 100 |
| 0.1 | 1.83 | 100 |
| 0.2 | 1.73 | 100 |
| 0.3 | 1.64 | 100 |
| 0.4 | 1.55 | 100 |
| 0.5 | 1.46 | 100 |
| 0.6 | 1.38 | 100 |
| 0.7 | 1.3 | 100 |
| 0.8 | 1.23 | 100 |

# References

[1] Marsal, J.-R., Ferreira-González, I., Bertran, S., Ribera, A., Permanyer-Miralda, G., García-Dorado, D., & Gómez, G. (2017). The Use of a Binary Composite Endpoint and Sample Size Requirement: Influence of Endpoints Overlap. American Journal of Epidemiology, 185(9), 832–841.

[2] Stone, G. W., Ellis, S. G., Cox, D. A., Hermiller, J., O'Shaughnessy, C., Mann, J. T., Turco, M., Caputo, R., Bergin, P., Greenberg, J., Popma, J. J., & Russell, M. E. (2004). A Polymer-Based, Paclitaxel-Eluting Stent in Patients with Coronary Artery Disease. New England Journal of Medicine, 350(3), 221–231.

[3] Stone, G. W., Ellis, S. G., Cannon, L., Mann, J. T., Greenberg, J. D., Spriggs, D., O'Shaughnessy, C. D., DeMaio, S., Hall, P., Popma, J. J., Koglin, J., Russell, M. E., & TAXUS-V Investigators, for the. (2005). Comparison of a Polymer-Based Paclitaxel-Eluting Stent With a Bare Metal Stent in Patients With Complex Coronary Artery Disease. JAMA, 294(10), 1215.
